# Soil organic matter and soil structure changes with tillage practices and straw incorporation in a saline-sodic soil

**DOI:** 10.3389/fpls.2025.1681651

**Published:** 2025-11-10

**Authors:** Azhar Ali Laghari, Quart-ul-ain Abro, Asma Leghari, Akash Kumar, Lata Kumari, Barkat Ali Nindwani, Sadia Gull

**Affiliations:** 1 College of Resources Environment, Shanxi Agricultural University, Taigu, Shanxi, China; 2 Business School, University of International Business and Economics, Beijing, China; 3 Institute of Clean Coal Technology, East China University of Science and Technology, Shanghai, China; 4 School of Civil Engineering, Guangzhou University, Guangzhou, China; 5 School of Chemical Engineering of Technology, Tianjin University, Tianjin, China; 6 Department of Farm Power and Machinery, Faculty of Agricultural Engineering, Sindh Agriculture University, Tandojam, Pakistan; 7 Department of Life Sciences, Western Caspian University, Baku, Azerbaijan

**Keywords:** tillage practices, straw incorporation, soil aggregation, water stableaggregates, saline sodic soil

## Abstract

Soil salinity and sodicity pose significant challenges to sustainable agriculture by adversely affecting soil properties, crop growth, and yield. The study was conducted in an agricultural field located in Khipro, within the Sanghar district of Sindh Province, Pakistan, to assess the impact of tillage practices and wheat straw incorporation on the organic matter content and structural properties of saline-sodic soil. Field experiments were conducted under shallow (ST) and deep tillage (DT) systems, a conventional practice (NPK fertilizer with gypsum, CK), a control (no straw or gypsum, CTRL), and three wheat straw application rates of 3, 7, and 10 Mg ha^−^¹ and gypsum application rates corresponding to 25%, 50%, and 75% of the gypsum requirement (GR). After two years, soil organic matter (SOM), water-stable aggregates (WSA), mean weight diameter (MWD), and aggregate stability (AS) were significantly improved (P ≤ 0.05) in treatments with incorporated wheat straw. Compared with the control, the combined application of deep tillage, 10 Mg ha^−^¹ straw, and 75% gypsum increased soil organic matter by approximately 35%, water-stable aggregates (>0.25 mm) by 12–18%, aggregate stability by 42%, and mean weight diameter by 31%. Improvements were more pronounced in the upper 0–15 cm soil layer than in deeper layers. Enzymatic activities, including dehydrogenase, urease, and alkaline phosphatase, also increased by 28–46% under the same treatment, indicating enhanced microbial activity and nutrient cycling. These findings demonstrate that integrating straw incorporation with deep tillage and gypsum amendment is an effective management practice to improve soil structure, organic matter content, and biological activity in saline-sodic soils under semi-arid conditions.

## 1 Introduction

The nation of Pakistan has an area of 0.80 × 10^6^ km^2^, of which almost 29% is cropland (0.23 × 10^6^ km^2^). Of this, 29% (66.7 × 10^3^ km^2^) currently suffers from salinity and sodicity, and this expanse is rapidly increasing (GOP, 2010; Khan, 1998). This increasing salinity/sodicity has reduced the average crop yield by >50% (Bray et al., 2000).

Soil salinity and sodicity constitute critical environmental constraints to the sustainability of agricultural systems, exerting deleterious effects on soil physicochemical properties, crop physiological development, and overall agronomic yield (Okur, 2002; Devkota et al., 2015). Soil salinity and sodicity pose significant challenges to agricultural systems by compromising soil integrity and crop productivity. High salinity levels disrupt soil structure, leading to increased vulnerability to erosion by wind and water (Zamani and Mahmoodabadi, 2013; Arjmand Sajjadi and Mahmoodabadi, 2015). Additionally, excessive salt accumulation impairs soil water permeability, hindering effective water movement through the soil profile (Mari et al., 2011). In terms of crop performance, elevated salinity heightens plant susceptibility to physiological stress and injury. It disrupts critical metabolic processes, which in turn limits the uptake and assimilation of essential nutrients, ultimately reducing nutrient use efficiency (Parida and Das, 2005). These adverse effects manifest as diminished plant growth and reduced crop yields, posing challenges to sustainable agricultural production (Katerji et al., 2009; Semiz et al., 2014).

Saline-sodic soils often exhibit compromised structural integrity and diminished soil organic matter (SOM) content, primarily due to the dispersive effects of sodium. This element actively disrupts soil aggregates and indirectly alters their size distribution and stability (Bronick and Lal, 2005; Moghimi et al., 2012). Research by Kantrikrom et al. (2020) indicates that higher sodium concentrations in soil correlate with reduced aggregate size and stability, exacerbating soil degradation. Furthermore, conventional tillage practices can exacerbate these issues by diminishing soil water retention and further destabilizing aggregate structure (Mele and Crowley, 2008). As a result, these combined factors contribute to a significant decline in crop productivity, posing challenges for sustainable agricultural management.

Tillage practices play a decisive role in determining soil physical and biological conditions. Appropriate tillage improves root penetration, enhances soil aeration, and promotes the formation of stable aggregates, whereas intensive or repeated tillage can accelerate the decomposition of soil organic matter and disrupt aggregate stability (Aase and Pikul Jr., 1995; Six et al., 2000). The type of crop grown also influences soil structure and organic matter turnover through variations in root biomass, residue quality, and rhizosphere activity. Deep-rooted crops such as wheat and maize promote aggregation by releasing root exudates and contributing organic residues to the soil matrix (Xing et al., 2022). In addition, soil texture strongly governs the response of saline-sodic soils to management interventions. Fine-textured soils, particularly those rich in clay, exhibit greater cation exchange capacity and aggregate-forming potential but are more prone to dispersion under high sodium concentrations (Warrence et al., 2002). Enzymatic activities, including dehydrogenase, urease, and phosphatase, are critical biochemical indicators of soil health. These enzymes mediate the cycling of key nutrients such as nitrogen, phosphorus, and carbon, and their activities reflect the biological recovery of salt-affected soils following amendments and residue incorporation (Rao et al., 2010; Singh et al., 2018). Therefore, understanding how tillage, cropping systems, soil texture, and enzyme activities collectively influence soil structure and SOM dynamics is essential for designing effective reclamation strategies in saline-sodic environments.

In numerous regions globally, strategic agricultural practices such as tillage, irrigation, crop selection, and the application of chemical or fertilizer amendments have emerged as critical tools for rehabilitating salt-affected soils and boosting crop yields (Jordan et al., 2004). The incorporation of organic matter, such as farmyard manure, crop residues, or compost, into saline soils is a widely adopted approach to mitigate the detrimental impacts of salinity on agricultural ecosystems. These organic amendments facilitate favorable physico-chemical transformations in salt-affected soils, significantly enhancing their fertility and productivity (Tahir et al., 1991; Gaffar et al., 1992; Liang et al., 2005). Research by Benbouali et al. (2013) further illustrates that organic inputs markedly improve the size and stability of soil aggregates in saline environments, although the efficacy of these interventions is influenced by the degree of salinity or sodicity present in the soil.

The integration of straw into saline-sodic soils offers substantial improvements to both their physical and chemical characteristics, fostering enhanced soil health and functionality. Physically, straw incorporation reduces bulk density (ρ), increases porosity (ϕ), enhances hydraulic conductivity (k_sat_), and mitigates clay dispersion, thereby improving soil structure and water movement (Hussain et al., 1998). Chemically, it contributes to lowering electrical conductivity (EC_e_), moderating soil pH, and reducing the sodium adsorption ratio (SAR), which collectively alleviate the adverse effects of salinity and sodicity (Hussain et al., 1998). Moreover, straw serves as a vital source of soil organic matter (SOM), which plays a pivotal role in enhancing key soil properties. These include improved nutrient availability, greater water retention capacity, increased cation exchange capacity, better soil reaction, reduced erosion, and enhanced capacity for pollution remediation (Ibrahim et al., 2011, 2012). By addressing these critical soil attributes, straw incorporation emerges as a promising and sustainable strategy for mitigating soil salinity and preventing its recurrence, offering a practical approach to restoring and maintaining soil productivity in salt-affected environments (Sarwar et al., 2008; Ahn et al., 2010; Sarwar et al., 2011).

Soil enzymatic activities are essential for nutrient cycling, ensuring that vital nutrients are made available to both plants and soil microbes (Ai et al., 2015). The microbial community in the soil is widely recognized as a key indicator of soil health and quality. The application of soil amendments has been shown to enhance enzymatic activity, contributing to increased crop yields (Jia et al., 2005; Singh, 2016). The structure and function of soil microbial communities are regulated by pH-dependent mechanisms, influencing their overall activity (Ai et al., 2015). To optimize restoration strategies for saline-alkaline soils, it is critical to gain a thorough understanding of how variations in soil physicochemical properties influence microbial composition and enzymatic functions. This knowledge is vital for developing more effective and sustainable soil management practices. The size, configuration, and resilience of soil aggregates significantly influence various agronomic and environmental processes (Diaz-Zorita and Grove, 2002; Ahmadi et al., 2011). Research by Sonnleitner et al. (2003) indicates that incorporating straw into soil enhances aggregate stability and improves other soil characteristics more effectively than farmyard manure. The practice of adding straw to soils, particularly in semiarid regions, has been recognized as a valuable strategy for increasing both the quantity and durability of soil aggregates (Zhang et al., 2014a). The effectiveness of straw incorporation in promoting aggregate formation is closely tied to both the quantity and quality of the straw used, as these factors play a critical role in the aggregation process (Lynch and Bragg, 1985). However, most existing studies have primarily focused on the use of organic amendments to enhance soil physicochemical properties, rather than directly targeting the reclamation of saline or sodic soils. Despite all the efforts made on a global scale, the expanse of soils suffering from salinity/sodicity is increasing (Bello et al., 2021; Sundha et al., 2020).

It is also important to note that the effectiveness of soil conservation and reclamation practices varies considerably across climatic zones. In arid and semi-arid regions such as Sindh, Pakistan, limited rainfall, high evaporation, and low organic matter inputs constrain the accumulation and stabilization of soil organic matter, making soil structure formation and restoration more challenging than in humid regions (Klemmedson, 1989; Heckman et al., 2023). Conversely, in humid climates, higher organic matter turnover and greater biological activity can enhance aggregate formation and the efficiency of conservation practices (Steffens et al., 2009; Dlamini et al., 2016). Therefore, understanding how tillage and straw incorporation influence saline-sodic soil properties under semi-arid conditions is essential for developing region-specific management strategies. However, the combined effects of gypsum amendment, tillage intensity, and straw incorporation on the mechanisms of soil organic matter stabilization and aggregate formation under saline-sodic conditions remain poorly understood (Shepherd and Walsh, 2002). Understanding these interactions is essential to reveal how integrated management practices improve soil structure and resilience.

We propose that integrating straw application with varied tillage methods and tailored gypsum amendments will enhance the structural integrity of saline-sodic soils while mitigating their salinity and sodicity challenges. Accordingly, the objectives of this study were to (i) assess the effects of different tillage practices and straw incorporation on soil organic matter (SOM) content and soil structural stability in saline-sodic conditions; (ii) determine the influence of these practices on soil characteristics and enzymatic properties; and (iii) identify the most effective management combination for improving soil health and productivity under semi-arid saline-sodic environments. To achieve these objectives, a field experiment was conducted on a farmer’s land in Taluka Khipro, Sindh, Pakistan, to evaluate the combined effects of tillage practices, straw incorporation, and gypsum application on SOM and the structural properties of saline-sodic soil.

## 2 Materials and methods

### 2.1 Experimental location

**Figure 1:**
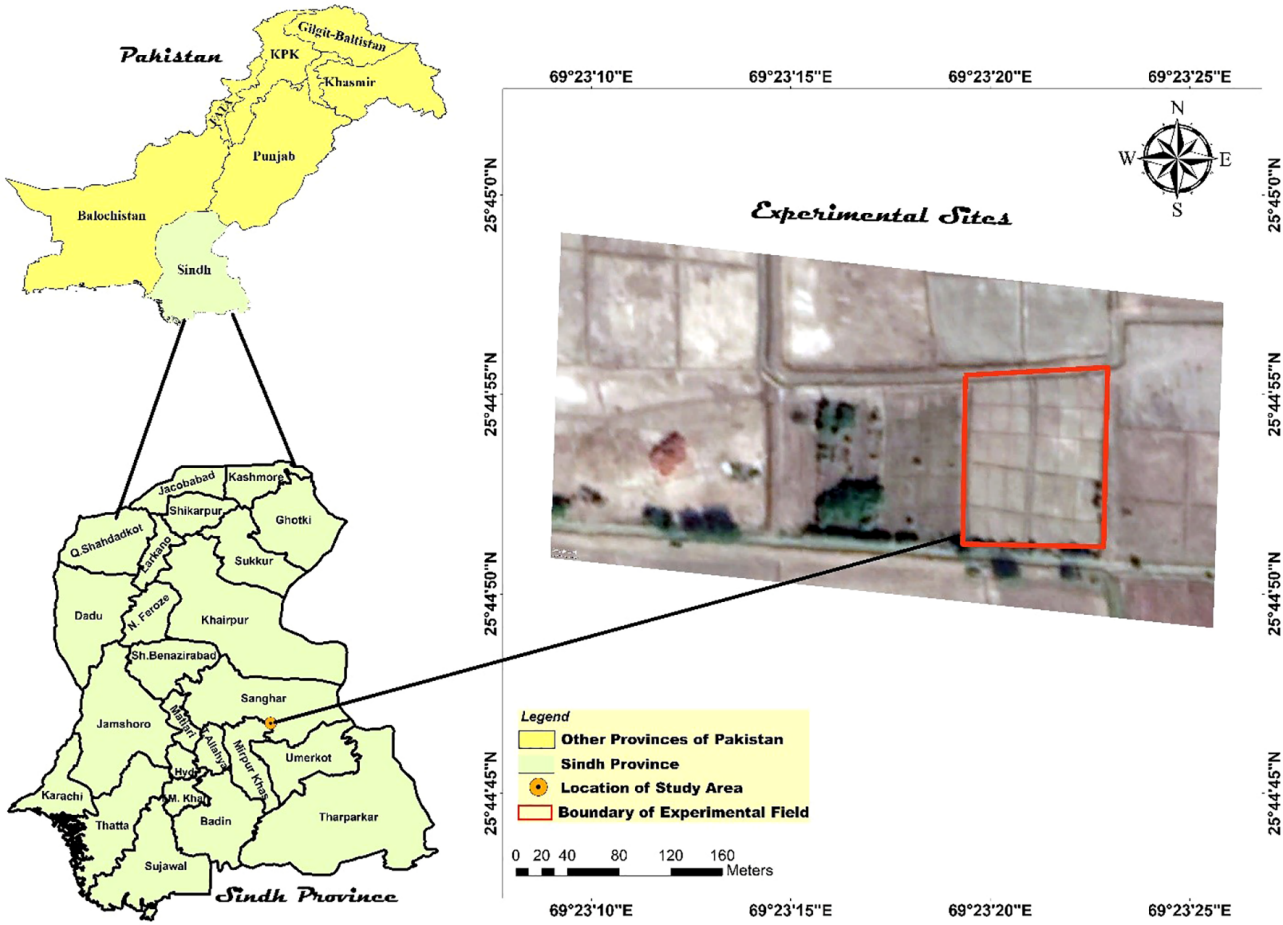
Spatial localization of the study site: The map illustrates the precise geographical coordinates of the experimental field utilized in this research.

The current study was conducted on an agricultural field situated on a farmer’s land in the Khipro region of Sanghar District, Sindh, Pakistan (latitude 25°45’0” N, longitude 69°23’15” E, elevation 13 meters above mean sea level; see **Figure 1**).

For over two decades, the experimental site remained unproductive due to challenges with soil salinity and sodicity, compounded by a lack of available water. Soil analyses, conducted following the USDA particle size classification method (Bouyoucos, 1927), and determined the soil across all treatment plots to be a silt loam. According to the FAO (2006) classification system, it was further identified as a Haplic Yermosol. These methods are widely used for characterising the physical properties and classification of arid-region soils (Gee and Bauder, 1986; FAO, 2006). The physical characteristics of the soil at the study site are detailed in **Table 1**.

**Table 1:** Soil depth, particle size distribution, textural class, electrical conductivity of soil saturation extract (dS m^-1^), soil pH, sodium absorption ratio (SAR), exchangeable sodium percentage (ESP).

Soil depth (cm)	Particle size distribution			Textural c lass	ECe (dS m^-1^)	pH	SAR	ESP
	Sand	Silt	Clay					
0-15	24.8	60.2	15	Silty loam	9.9	6.5	18.9	22.1
15-30	25.1	59.4	15.5	Silty loam	9.7	7.1	18.9	21.9
30-45	23.8	61.1	15.1	Silty loam	9.3	6.9	21.9	23.7

**Figure 2:**
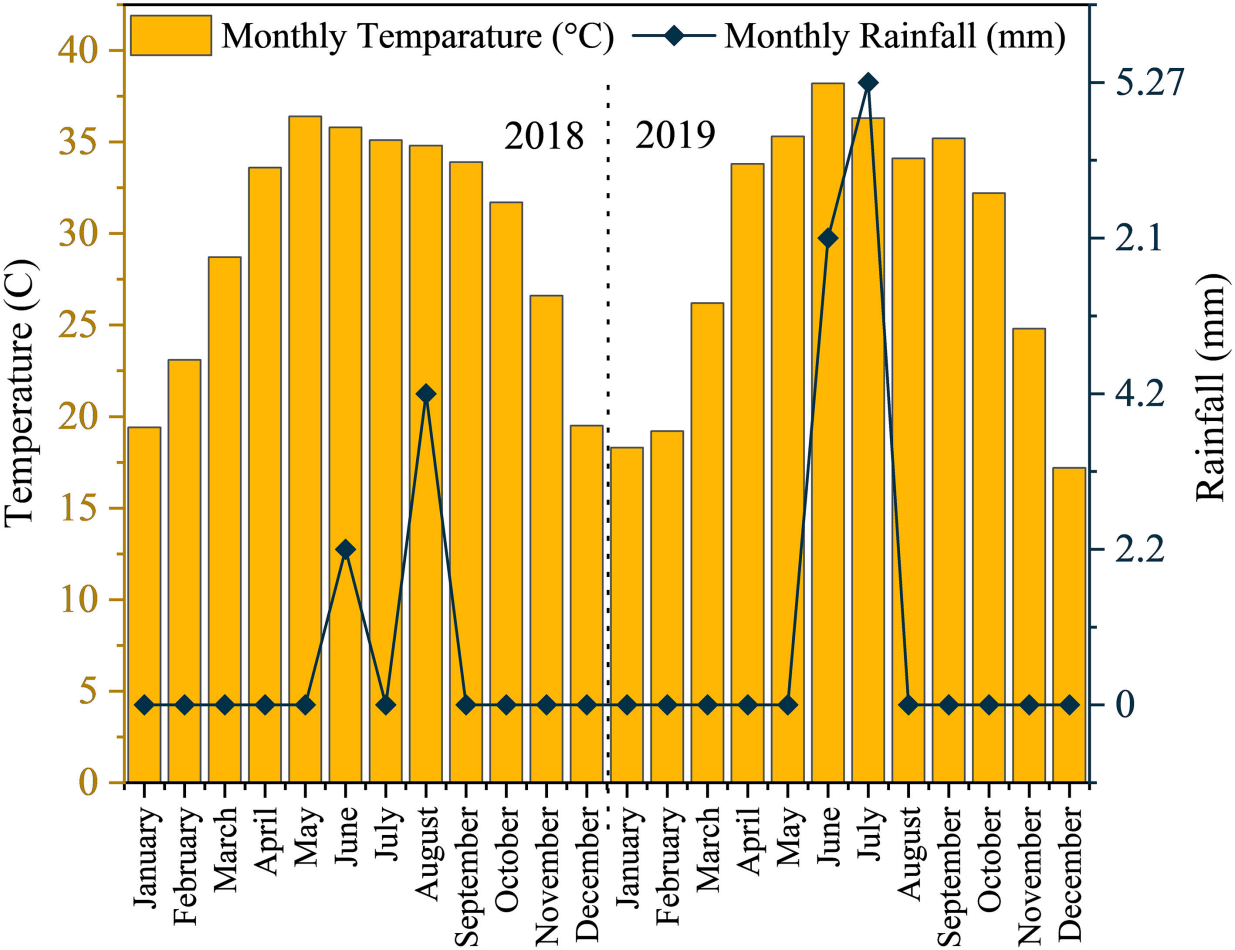
Monthly average temperature and monthly cumulative rainfall data for the experimental site during 2018-2019.

The average monthly minimum and maximum temperatures recorded were 18.21°C and 41.55°C in 2018, and 16.90°C and 40.57°C in 2019 (**Figure 2**). In terms of rainfall, the average monthly minimum and maximum values were 2.2 mm and 4.2 mm in 2018, and 2.1 mm and 5.2 mm in 2019, respectively (Abbas et al., 2018).

### 2.2 Experimental plan

The experiment was laid out using a randomized complete block design (RCBD) with four replications, and the treatments were assigned as follows:

- Tillage
  ▪ shallow tillage (ST) performed up to a depth of 0.10 meters using two passes with a disk harrow
  ▪ deep tillage (DT), Moldboard ploughing to a depth of 0.25 meters, followed by two passes with a disk harrow, similar to the shallow tillage treatment.
- Wheat straw amendments
  ▪ 3 Mg ha^-1^ (WS3)
  ▪ 7 Mg ha^-1^ (WS7)
  ▪ 10 Mg ha^-1^. (WS10)
- Gypsum amendments
  ▪ 25% of gypsum requirement (GR25)
  ▪ 50% of gypsum requirement (GR50)
  ▪ 75% of gypsum requirement. (GR75)
- Conventional practice (CK) with no straw, only NPK and gypsum,
- Control (CTRL) No application of straw or gypsum,

The experimental field (66 m × 520 m) was accordingly divided into 104 plots (26 × 4) (15 m × 20 m each) (**Figure 1**). Wheat straw for Kharif crops and rice straw for Rabi crops were chopped into 50 mm pieces (Zhang et al., 2014b) and the three rates of straw were mixed with the soil using the specified tillage practices during 2018–2019.

The gypsum requirement was calculated as described in Equation (1) (Nan et al., 2016):

(1) $$\text{GR}=0.344\cdot \text{ρ}\cdot \text{d}\cdot ({\left[{\text{Ca}}^{2+}\right]}_{\text{g}}-{\left[{\text{Ca}}^{2+}+{\text{Mg}}^{2+}\right]}_{\text{f}})$$

Where,

GR Is the soil gypsum obligation (Mg ha^-1^)
${\left[{\text{Ca}}^{2+}\right]}_{\text{g}}$ Is the calcium ion (Ca²^+^) concentration in a saturated gypsum solution (me L^-1^)
${\left[{\text{Ca}}^{2+}+{\text{Mg}}^{2+}\right]}_{\text{f}}$ Is the Ca^2+^ + Mg^2+^ content of the filtrate (me L^-1^)
d Is the soil depth (m)
ρ Is the soil bulk density (Mg m^-3^)

Irrigation water was applied when the soil water content (*θ*) dropped below 60% of the field capacity (Ministry of Food, Agriculture and Livestock, 1997). The *θ* at 60% depletion ( $\theta_{o}$) was determined using Equations (2) and (3) (Hansen et al., 1979):

(2) $$SMD=\theta_{fc}-\theta_{o}$$

(3) $$\theta =100\cdot \frac{W_{w}-W_{d}}{W_{d}}$$

Where,

*SMD* Is the soil moisture deficit,
θ Is the measured soil water content (%),
$\theta_{fc}$ Is the soil water content at field capacity (%),
$\theta_{o}$ Is the water content at 60% depletion,
$W_{d}$ Is the weight of dry soil (g),
$W_{w}$ Is the weight of moist soil (g).

### 2.3 Irrigation regime and water quality

Irrigation was applied through a surface flood irrigation system using groundwater pumped from a nearby tube well, a common practice in the Khipro region. The irrigation schedule was managed based on soil moisture monitoring, with irrigation events occurring when soil water content dropped below 60% of field capacity, typically every 12–15 days during the cropping season. On average, 60–70 mm of water was applied per irrigation event. The chemical quality of the irrigation water was analyzed before each cropping season. The average electrical conductivity (ECw) of the irrigation water was 2.8 dS m^−^¹, pH was 7.6, and the sodium adsorption ratio (SAR) was 8.4, indicating moderately saline water according to FAO (Ayers and Westcot, 1985). This quality of irrigation water is typical of groundwater used for irrigation in semi-arid regions of Sindh, Pakistan, and can contribute to gradual salt accumulation if not managed with amendments such as gypsum.

### 2.4 Soil sampling and measurement

Before tillage and straw incorporation, as well as following the harvest of each crop, soil samples were obtained from depths of 0–0.15 m, 0.16–0.30 m, and 0.31–0.45 m at four randomly chosen locations within each experimental plot. These samples were combined to create a composite soil sample for each depth and plot, the sample was subsequently placed in clearly labeled aluminum containers and transported to the laboratory for analysis. In the laboratory, any litter, rock fragments, and surface crust were carefully removed from the samples. The soil was then air-dried at ambient room temperature for a period of 15 days to prepare it for further analysis.

Organic carbon content was determined using the Walkley and Black (1934) method. Soil organic carbon content (OC), expressed as a percentage (w/w) was calculated in Equation (4) (Nelson and Sommers, 1996):

(4) $$\text{OC}=100\cdot \frac{0.003\text{N}\cdot (\text{B}-\text{S})}{{\text{W}}_{\text{d}}}$$

where,

*B* Is the volume of standard 0.5 N Fe (NH_4_)_2_(SO_4_)_2_ required to titrate the blank (mL),
*N* Is the normality of the standard Fe (NH_4_)_2_(SO_4_)_2_ solution (0.5 N),
*S* Is the volume of standard 0.5 N Fe (NH_4_)_2_(SO_4_)_2_ required to titrate the soil sample, and W_d_ is as defined above.

Organic carbon was then converted to soil organic matter content by multiplying by a factor of 1.72 (Mattingly, 1974).

To assess aggregate size distribution, air-dried soil samples were sieved through a series of nested sieves with mesh sizes of 32, 25, 12.5, 8, 2, 1.2, 0.5, 0.25, 0.15, and 0.015 mm. The amount of soil retained on each sieve was then collected and weighed to determine the distribution across size classes. Soil aggregate size distribution was then determined in Equation (5) by the Van Bavel (1949) method:

(5) $$MWD=\sum_{i=1}^{i=n}x_{i}w_{i}$$

Where,

$x_{i}$ Is the mean diameter of adjacent sieves,
$w_{i}$ Is the proportion (fraction) of total sample retained on the *i*^th^ sieve,
MWD Is the mean weight diameter of the average size of soil aggregates,
*n* Is the number of different sieves.

Aggregate stability was assessed using the wet sieving method described by Kemper and Rosenau (1986). A 50 g portion of air-dried soil, previously sieved through an 8 mm mesh, was placed on a nest of sieves with mesh sizes of 2.00, 1.00, 0.50, and 0.25 mm. The sieves were pre-soaked in distilled water for 30 minutes. Subsequently, the setup was immersed in distilled water and subjected to oscillation at a rate of 30 cycles per minute for 10 minutes. Water-stable aggregate stability (AS) was then calculated in Equation (6), following the method outlined by Piccolo and Mbagwu (1999).

(6) $$\text{AS}=100\cdot (\frac{{\text{M}}_{\text{WSA}>0.05{}^{\circ}\text{mm}}-{\text{M}}_{\text{sand}}}{{\text{M}}_{\text{soil}}-{\text{M}}_{\text{sand}}})$$

where,

$M_{sand}$ is the total mass of sand (0.5-2.00 mm),
$M_{soil}$ is the total mass of soil,
${\text{M}}_{\text{WSA}>0.5\text{ mm}}$ is the mass of water stable aggregates.

### 2.5 Soil enzymatic activities

In their 1985 study, Breuil and Saddler (1985) described a method employing 3,5-dinitrosalicylic acid to measure the activity of soil cellulase (S-CL), providing a reliable approach for assessing this enzyme’s function in soil ecosystems. The activity of soil catalase (S-CAT) was evaluated using a technique outlined by Alef and Nannipieri in 1995, which has been widely adopted for its precision in quantifying catalase activity. For the determination of soil alkaline phosphatase (S-ALP), the p-nitrophenyl phosphate method, as detailed by Tabatabai and Bremner in 1969, was utilized, offering a robust protocol for evaluating phosphatase activity in soil samples. Additionally, the activity of soil urease (S-UE) was assessed through a method developed by Kandeler and Gerber in 1988, which remains a standard for studying urease dynamics in soil environments.

### 2.6 Statistical analysis

A multifactor analysis of variance (ANOVA) was performed using the Statistics 8.1 software package (Analytical Software, 2005) to evaluate the main and interaction effects of tillage (shallow vs. deep), gypsum application rates (25%, 50%, and 75% of the gypsum requirement), and straw incorporation levels (0, 3, 7, and 10 Mg ha^−^¹) on soil organic matter content, water-stable aggregates, aggregate stability, and mean weight diameter. Interaction effects among the factors (tillage × gypsum, tillage × straw, straw × gypsum, and tillage × gypsum × straw) were also tested. To ensure the validity of the ANOVA results, the normality of residuals was assessed for all variables using the Shapiro–Wilk test, which confirmed that residuals followed a normal distribution (p > 0.50). When significant treatment effects were detected, mean comparisons were performed using Duncan’s multiple range test (DMRT) at the 0.05 probability level to separate differences among means. Detailed ANOVA tables presenting F-values, degrees of freedom, and significance levels for all main and interaction effects are provided in the **Supplementary Material** (**Appendices A–G**).

## 3 Results

The applied treatments significantly influenced soil organic matter, the formation of water-stable aggregates, mean weight diameter, and overall aggregate stability, demonstrating substantial effects on soil structural properties. (*P ≤* 0.01) (**Supplementary Table S1**). The interactions of treatment × tillage, treatment × year and treatment × tillage × year were significant (*P ≤* 0.01) for water stable aggregates, mean weight diameter and aggregate stability (**Supplementary Tables S2** and **S3**) and for organic matter at a depth of 0-0.15 m, whereas the interactions for treatment × tillage, treatment × year and treatment × tillage × year were not significant (*P >* 0.05) for SOM at a depth of 0.16-0.45 m.

### 3.1 Soil organic matter (SOM)

**Figure 3:**
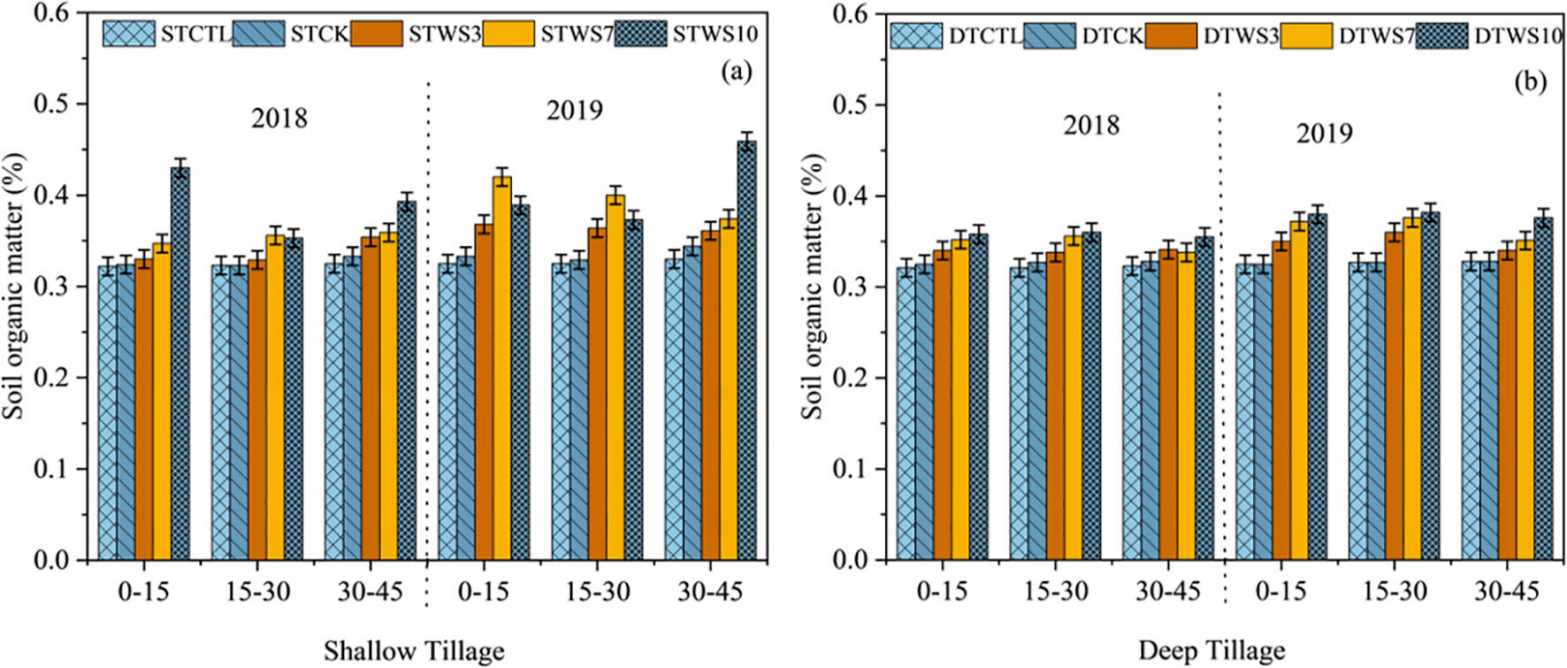
Soil organic matter under **(a)** shallow and **(b)** deep tillage practices and straw incorporation rates in saline sodic soil. ^1^ST_CTRL_: Control treatment under shallow tillage; ST_CK_: Conventional tillage under shallow tillage; ST_WS3_: Straw incorporation at 3 Mg ha^−^¹ under shallow tillage; ST_WS7_: Straw incorporation at 7 Mg ha^−^¹ under shallow tillage; STWS10: Straw incorporation at 10 Mg ha^−^¹ under shallow tillage. ^2^ DT_CTRL_: Control treatment under deep tillage; DT_CK_: Conventional tillage under deep tillage; DT_WS3_: Straw incorporation at 3 Mg ha^−^¹ under deep tillage; DT_WS7_: Straw incorporation at 7 Mg ha^−^¹ under deep tillage; DTWS10: Straw incorporation at 10 Mg ha^−^¹ under deep tillage. ^3^ Values are presented as mean ± standard error.

After two years, under straw-amended treatments, SOM was 6.89% - 15.93% greater under shallow tillage (ST_WS3 -_ ST_WS10_), and 6.82% - 9.36% greater under deep tillage (DT_WS3_ - DT_WS10_), than under non-amended treatments (ST_CK_ and DT_CK_ and ST_CTRL_ and DT_CTRL_) (**Figure 3a, b**: **Supplementary Table S4**). The SOM showed a significant increase with the rate of straw amendment, following the order ST_WS10_ > DT_WS10_ > ST_WS7_ >DT_WS7_ > ST_WS3_ > DT_WS3_. The soil organic carbon (SOC) was 3.63%, 3.95%, 4.56%, 1.5% and 1.18% greater under shallow tillage treatments (*i.e*., ST_WS3,_ ST_WS7,_ ST_WS10_, ST_CK_ and ST_CTRL_) than under the equivalent deep tillage treatments (*i.e*., DT_WS3,_ DT_WS7,_ DT_WS10,_ DT_CK_ and DT_CTRL_). The SOC increased significantly (*P* ≤ 0.05) with the passage of time. In 2019, SOC under shallow tillage was 3.63% to 4.56% greater than in 2018. while under deep tillage, SOC was 2.95% to 3.74% greater.

### 3.2 Water stable aggregates

**Figure 4:**
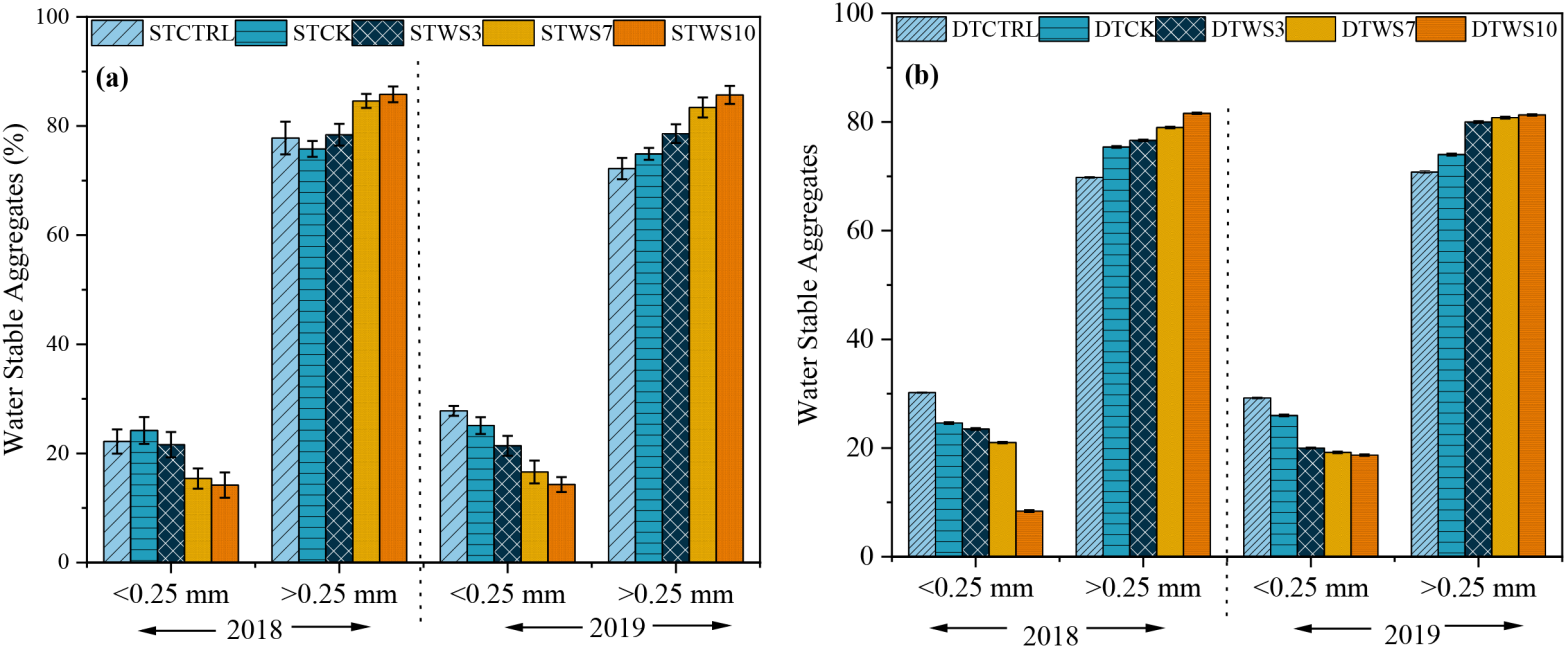
Water stable aggregates (%) under **(a)** shallow tillage (during 2018, 2019) and **(c,d)** deep tillage practices (during 2018, 2019) and straw incorporation rates in saline sodic soil. ^1^STCTRL: Control treatment under shallow tillage; STCK: Conventional tillage under shallow tillage; STWS3: Straw incorporation at 3 Mg ha^−^¹ under shallow tillage; STWS7: Straw incorporation at 7 Mg ha^−^¹ under shallow tillage; STWS10: Straw incorporation at 10 Mg ha^−^¹ under shallow tillage. ² DTCTRL: Control treatment under deep tillage; DTCK: Conventional tillage under deep tillage; DTWS3: Straw incorporation at 3 Mg ha^−^¹ under deep tillage; DTWS7: Straw incorporation at 7 Mg ha^−^¹ under deep tillage; DTWS10: Straw incorporation at 10 Mg ha^−^¹ under deep tillage. ³ Values are expressed as mean ± standard error.

After two years, under straw-amended treatments, WSA were 1.71%-1.92% greater under shallow tillage (ST_WS3 -_ ST_WS10_), and 6.74%-8.07% greater under deep tillage (DT_WS3_ - DT_WS10_), than under non-amended treatments (ST_CK_ and DT_CK_ and ST_CTRL_ and DT_CTRL_) (**Figures 4a-d**: **Supplementary Table S5**). The WSA significantly increased with the rate of straw amendment. Compared to the ST_WS3_ and DT_WS3_ treatments, high WSA values were found under ST_WS10_ and DT_WS10_**,** followed by ST_WS7_ and DT_WS7_. The WSA was 3.07, 2.65, 5.51, 1.75 and 1.68% greater (absolute) under shallow tillage treatments (*i.e*., ST_WS3,_ ST_WS7,_ ST_WS10_, ST_CK_ and ST_CTRL_) than under equivalent deep tillage treatments (*i.e*., DT_WS3,_ DT_WS7,_ DT_WS10,_ DT_CK_ and DT_CTRL_). The WSA significantly (*P ≤* 0.05) increased from the first to the second year, being 1.71-7.43% and 6.74-8.48% greater under shallow and deep tillage practices, respectively, in 2019 than in 2018.

### 3.3 Mean Weight Diameter

**Figure 5:**
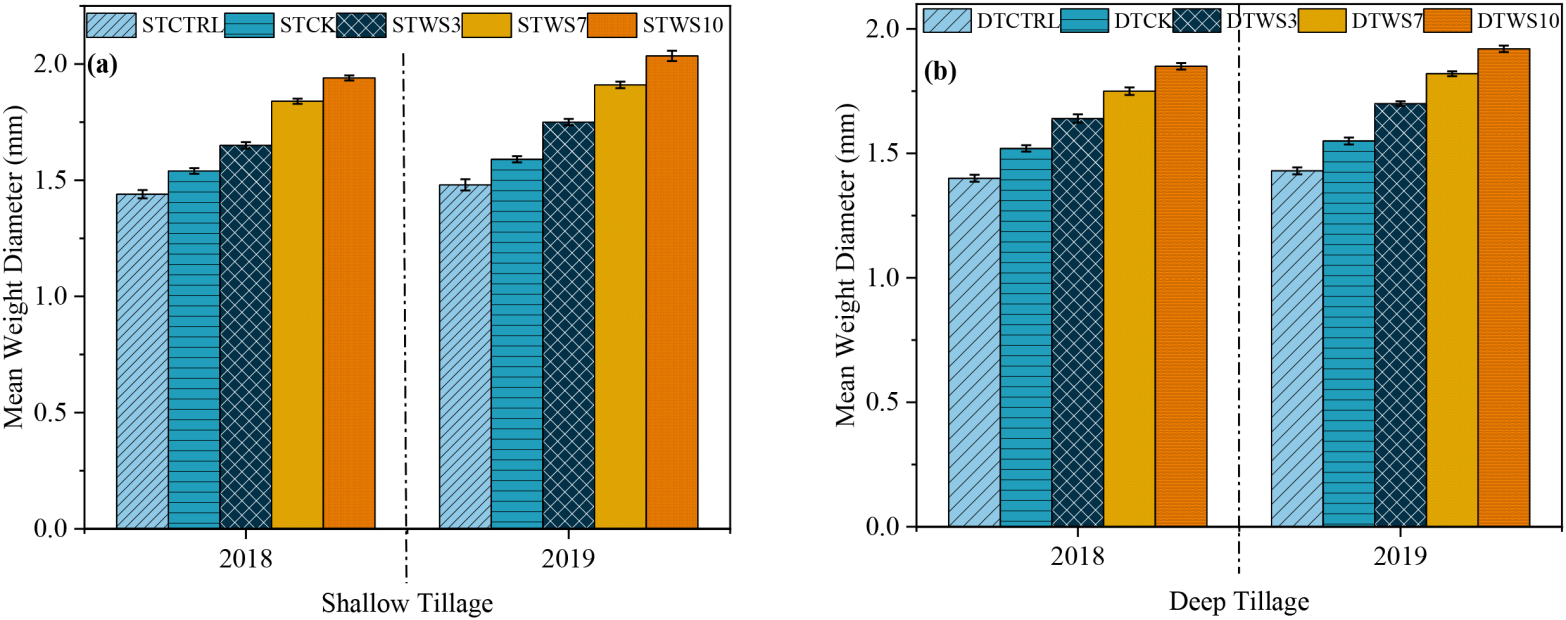
Mean weight diameter (mm) under **(a)** shallow and **(b)** deep tillage practices and straw incorporation rates in saline sodic soil. ^1^ST_CTRL_ control treatment under shallow tillage; ST_CK_ conventional tillage under shallow tillage; ST_WS3_ straw incorporation at the rate of 3 Mg ha^-1^ under shallow tillage; ST_WS7_ straw incorporation at the rate of 7 Mg ha^-1^ under shallow tillage; ST_WS10_ straw incorporation at the rate of 10 Mg ha^-1^ under shallow tillage. ^2^DT_CTRL_ control treatment under deep tillage; DT_CK_ conventional tillage under deep tillage; DT_WS3_ straw incorporation at the rate of 3 Mg ha^-1^ under deep tillage; DT_WS7_ straw incorporation at the rate of 7 Mg ha^-1^ under deep tillage; DT_WS10_ straw incorporation at the rate of 10 Mg ha^-1^ under deep tillage. ^3^Mean ± standard error.

After two years, under straw-amended treatments, MWD was 1.71%-1.91% greater under shallow tillage (ST_WS3 -_ ST_WS10_), 1.64%-1.84% greater under deep tillage (DT_WS3_ - DT_WS10_), than under non-amended treatments (ST_CK_ and DT_CK_ and ST_CTRL_ and DT_CTRL_) (**Figures 5a, b**: **Supplementary Table S6**). The MWD increased suggestively (*P* ≤ 0.05) with the rate of straw incorporated into the soil, ranking in the order of ST_WS10_ > DT_WS10_ >ST_WS7_ >DT_WS7_ > ST_WS3_ > DT_WS3_. The MWD was 0.1%, 0.065%, 0.095%, 0.05% and 0.04% greater under shallow tillage treatments (*i.e*., ST_WS3,_ ST_WS7,_ ST_WS10_, ST_CK_ and ST_CTRL_) than under the matching deep tillage treatments. The MWD increased significantly (*P* ≤ 0.05) during the study period. Under both shallow and deep tillage practices, the MWD was 0.1% to 0.095% and 0.07% to 0.075% greater, respectively, in 2019 than in 2018.

### 3.4 Aggregate stability

**Figure 6:**
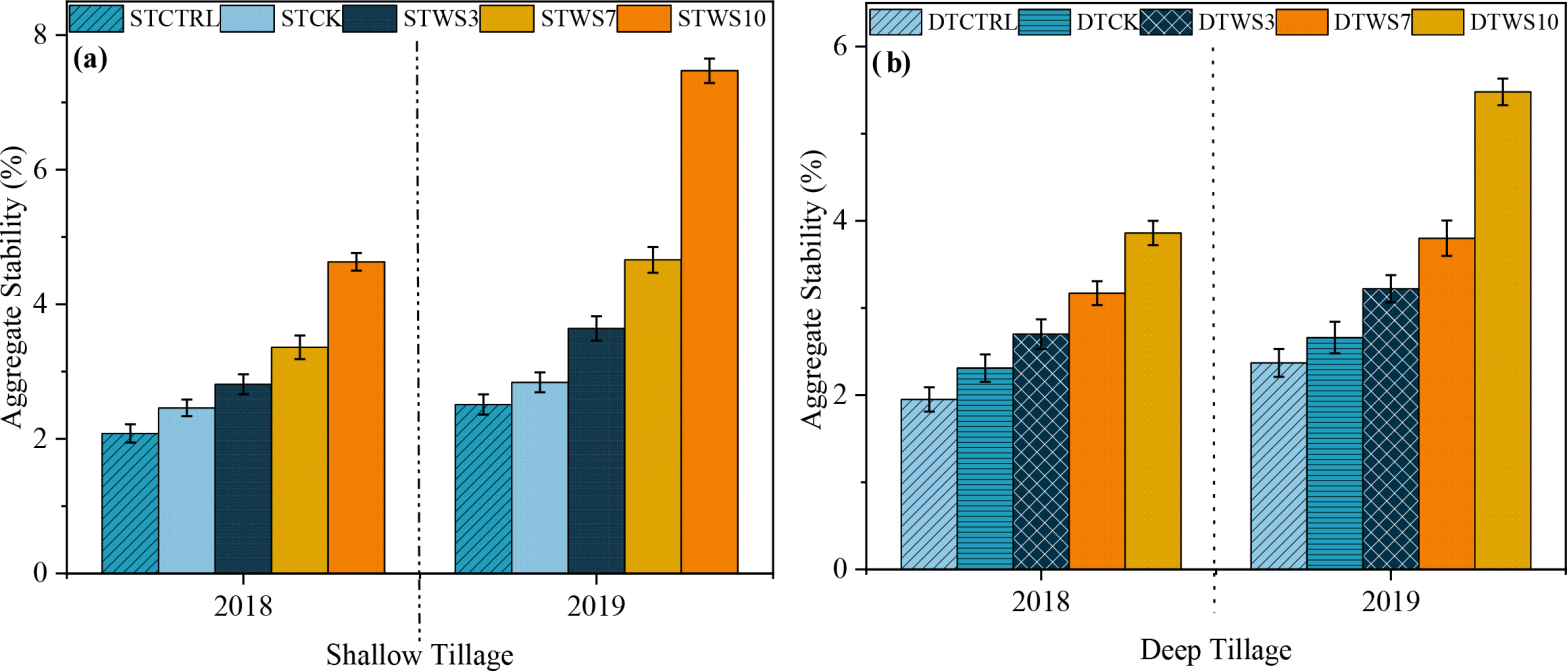
Aggregate stability under **(a)** shallow and **(b)** deep tillage practices and straw incorporation rates in saline sodic soil. ^1^ST_CTRL_ control treatment under shallow tillage; ST_CK_ conventional tillage under shallow tillage; ST_WS3_ straw incorporation at the rate of 3 Mg ha^-1^ under shallow tillage; ST_WS7_ straw incorporation at the rate of 7 Mg ha^-1^ under shallow tillage; ST_WS10_ straw incorporation at the rate of 10 Mg ha^-1^ under shallow tillage. ^2^DT_CTRL_ control treatment under deep tillage; DT_CK_ conventional tillage under deep tillage; DT_WS3_ straw incorporation at the rate of 3 Mg ha^-1^ under deep tillage; DT_WS7_ straw incorporation at the rate of 7 Mg ha^-1^ under deep tillage; DT_WS10_ straw incorporation at the rate of 10 Mg ha^-1^ under deep tillage. ^3^Mean ± standard error.

After two years, under straw-amended treatments, aggregate stability was 5.96%-11.68% greater under shallow tillage (ST_WS3 -_ ST_WS10_), 6.22%-12.86% greater under deep tillage (DT_WS3_ - DT_WS10_), than under non-amended treatments (ST_CK_ and DT_CK_ and ST_CTRL_ and DT_CTRL_) (**Figure 6a, b**: **Supplementary Table S7**). The AS increased significantly (*P* ≤ 0.05) with the rate of soil straw amendment, ranking in the order: ST_WS10_ > DT_WS10_ >ST_WS7_ >DT_WS7_ > ST_WS3_ > DT_WS3_. The AS was 5.96%, 7.27%, 11.68%, 3.61% and 2.89% greater under shallow tillage treatments (*i.e*., ST_WS3,_ ST_WS7,_ ST_WS10_, ST_CK_ and ST_CTRL_) than the matching deep tillage treatments. Moreover, the AS increased significantly (*P* ≤ 0.05) over the study period. Under shallow and deep tillage practices, AS was 0.13% to 2.27% and 0.09% to 2.45% greater, respectively, in 2019 than in 2018.

### 3.5 Soil enzymatic activity under shallow tillage

**Figure 7:**
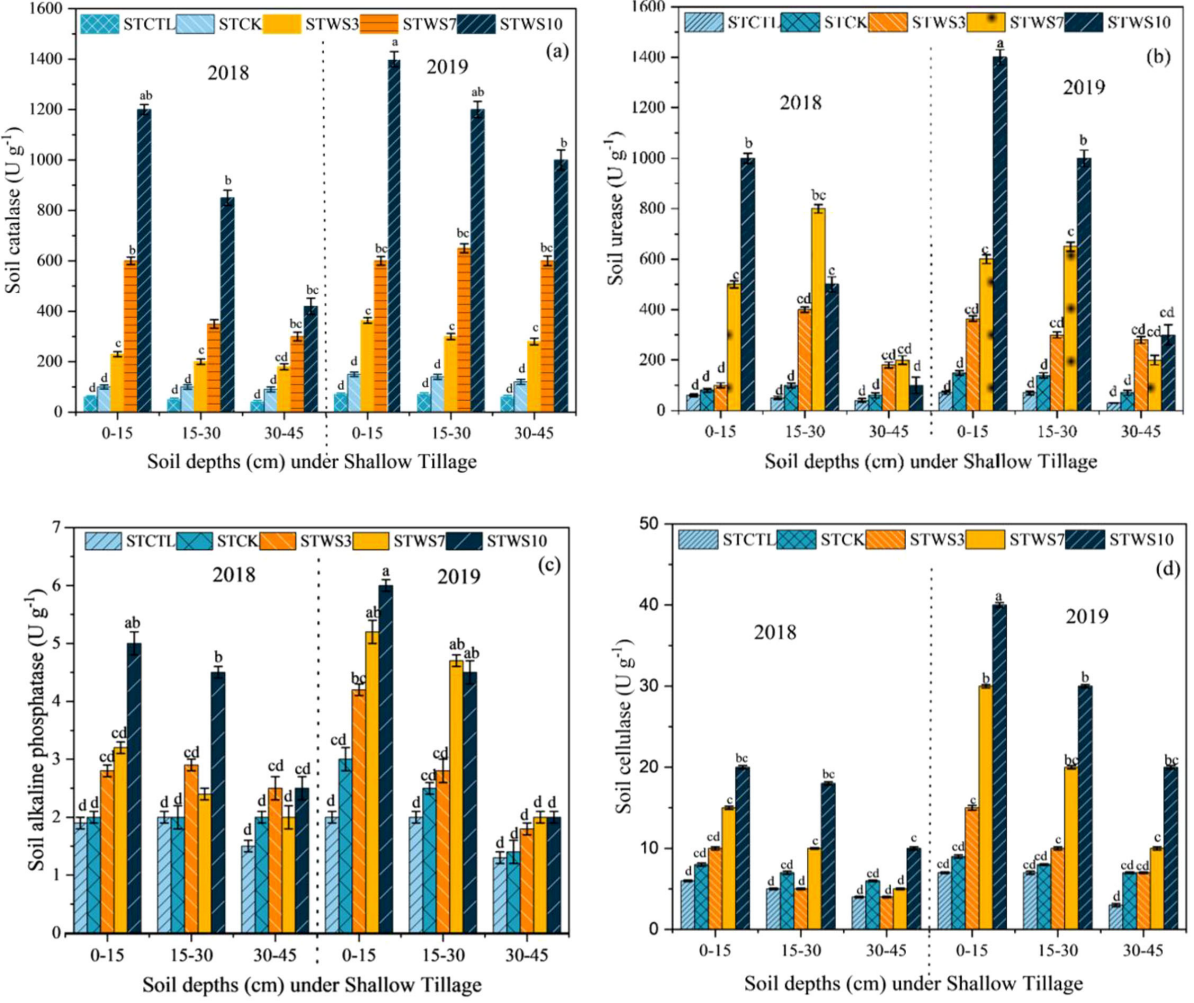
Enzyme activities of soil catalase, S-CAT **(a)** soil urease, S-UE **(b)** soil alkaline phosphatase, S-ALP **(c)** and soil cellulase, S-CL **(d)**. ST_CK_ conventional tillage under shallow tillage; ST_WS3_ straw incorporation at the rate of 3 Mg ha^-1^ under deep tillage; ST_WS7_ straw incorporation at the rate of 7 Mg ha^-1^ under deep tillage; ST_WS10_ straw incorporation at the rate of 10 Mg ha^-1^ under shallow tillage. The mean (± SE) of three replicates per treatment is utilized to represent the data and the differences in values among panels bearing similar letters were not statistically significant at a significance level of P ≤ 0.05.

The soil catalase activity (S-CAT) was significantly higher at 0–15 cm depth by STW10 straw management, followed by STW7 STCTR, STW3, STCK, and STCTR, respectively and the soil urease (S-URE), soil alkaline phosphatase (S-APH), and soil cellulase (S-CL) The tillage depth and straw management practices, along with their interactive effects, significantly influenced various activities in both years of the study. (2018-2019). Under the STW10 treatment, the soil S-CAT> S-URE > S-APH> S-CL activities at the 0–15 cm depth, and S-APH and S-URE activity at the 15–30 cm depth was increased, at depth 30–45 cm, all soil enzymatic were significantly decreased as compared STCTR by the Shallow tillage (ST) method. Soil S-CAT activity at the 0–15 cm depth was higher under the STW10 treatment in 2018 compared to 2019. Additionally, S-APH activity in the 15–30 cm soil layer increased across all treatments in both 2018 and 2019, relative to the CK treatment (**Figure 7a**). At the 30–40 cm depth, both S-CAT and S-CL activities were elevated under all treatments during 2018 and 2019 compared to the STCK treatment (**Figure 7**). Overall, S-CAT activity showed a gradual increase with the treatments STW3, STW7, and STW10, particularly under shallow tillage, and was notably higher than the STCTR treatment at the 0–10 cm depth.

### 3.6 Soil enzymatic activity under deep tillage

**Figure 8:**
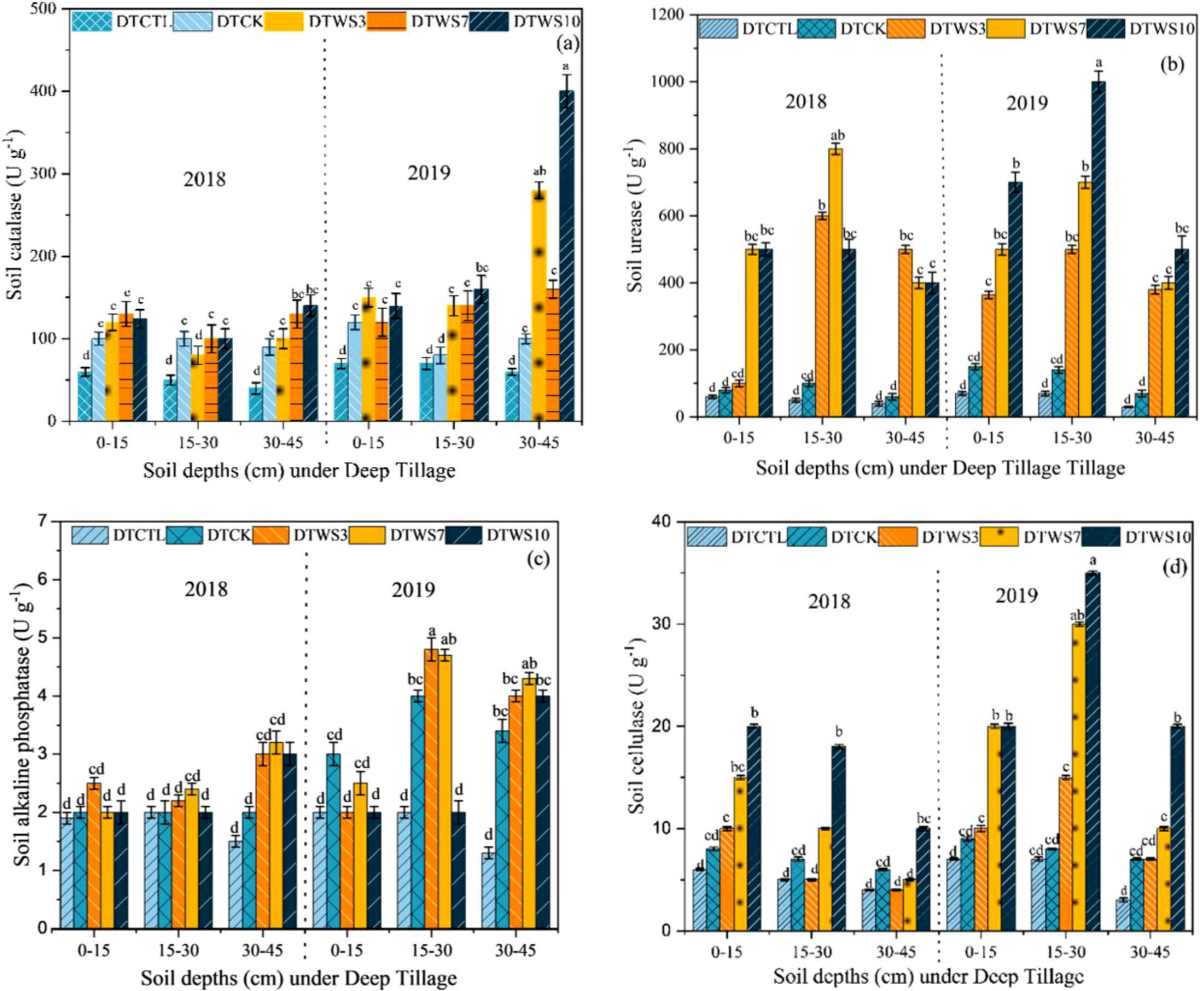
Enzyme activities of soil catalase, S-CAT **(a)** soil urease, S-UE **(b)** soil alkaline phosphatase, S-ALP **(c)** and soil cellulase, S-CL **(d)**. DT_CK_ conventional tillage under deep tillage; DT_WS3_ straw incorporation at the rate of 3 Mg ha^-1^ under deep tillage; DT_WS7_ straw incorporation at the rate of 7 Mg ha^-1^ under deep tillage; DT_WS10_ straw incorporation at the rate of 10 Mg ha^-1^ under deep tillage. The mean (± SE) of three replicates per treatment is utilized to represent the data and the differences in values among panels bearing similar letters were not statistically significant at a significance level of P ≤ 0.05.

Soil enzyme activities exhibited variations across different treatments. Specifically, S-CAT activity demonstrated a notable increase, at 30–45 cm depth by DT_WS10_ treatment and DT_WS3_ was slightly decreased from DT_WS10_ in 2019, whereas STW7 and DTCK showed no difference compared with DTCK at depth of 0–45 cm (**Figure 8a**). S-UE activity was highly affected at 0–15 cm, and 30–45 cm depths among all treatments, but at depth of 15–30 cm was increased with DT_WS10_ treatment in 2019 (**Figure 8b**). Moreover, during S-ALP activity no significant increase was observed in all treatments, but slighter increased at 15–30 cm in 2109 as compared to 2018 under DTWS10 and DT_WS7_ treatments (**Figure 8c**). The S-CL activity was increased in DT_WS10_ and DT_WS7_ followed by DT_WS3_ and DTCK and DTCTR (**Figure 8d**).

The summary of **Figure 8** shows that under deep tillage method enzymatic activities there was no significant difference at depth 0–15 cm among all treatments, whereas the S-CAT was higher at 30–45 cm. S-UE, S-ALP, and S-CL were higher at 15–30 cm depth by DT_WS10_ treatment.

## 4 Discussion

The current research found no statistically significant (P > 0.05) impact of gypsum application on soil organic matter (SOM), water-stable aggregates (WSA), mean weight diameter (MWD), or aggregate stability (AS) after a two-year period. These findings align with those reported by Kim et al. (2017), who determined that gypsum, when applied alone, did not significantly alter soil organic carbon (SOC) levels within soil aggregates compared to control treatments. However, treatments involving rice straw compost or a combination of gypsum and rice straw compost led to notable increases in SOC within soil aggregates, rising from an initial value of 1.09% to 1.66% in the gypsum plus rice straw compost treatment and 1.59% in the rice straw compost treatment alone.

In our research, after a two-year period, soil organic matter (SOM) exhibited a statistically significant increase (P ≤ 0.05) in treatments amended with straw compared to those without straw amendments. This finding aligns with the observations of Zhang et al. (2014a), who reported a notable enhancement in soil organic carbon (SOC) storage following three years of straw incorporation relative to control treatments. Additionally, SOC levels were found to be higher under shallow tillage compared to deep tillage practices. This outcome corroborates the findings of Tripathy and Singh (2004), who noted a progressive decline in SOC storage with increasing soil depth, attributed to reduced straw incorporation in deeper soil layers compared to the surface layers (0–0.20 m). Similarly, Prasad and Power (1991) and Rasmussen and Collins (1991) observed that straw incorporation was more pronounced in the topsoil than in deeper horizons, further supporting these trends.

The mechanism underlying these integrated treatments can be attributed to complementary physical, chemical, and biological processes. Incorporation of crop straw provides a continuous input of organic carbon, which fuels microbial growth and enzymatic activity. These microbial processes produce binding agents such as polysaccharides and humic substances that cement soil particles into stable aggregates (Six et al., 2000; Zhang et al., 2014). Gypsum addition supplies soluble Ca²^+^ ions that replace exchangeable Na^+^ on clay surfaces, reducing dispersion and promoting flocculation, which further enhances aggregate formation (Bronick and Lal, 2005; Liang et al., 2005). Tillage modifies the distribution of organic residues and improves the contact between soil particles, Ca²^+^, and microbial biomass, accelerating the stabilization of aggregates (Bronick and Lal, 2005). Under treatments without straw, the absence of organic binding agents limits microbial activity and aggregate cohesion, leading to lower organic matter retention and smaller mean weight diameter. Therefore, the combined application of straw and gypsum under appropriate tillage creates a synergistic effect: straw improves biological aggregation and carbon input, while gypsum improves the physicochemical environment for aggregate stability. Together, these processes explain the observed increase in soil organic matter, water-stable aggregates, aggregate stability, and mean weight diameter under integrated management practices.

Irrigation also played an essential role in modulating the effects of the integrated treatments on soil organic matter and structural stability. Adequate irrigation improved salt leaching from the root zone, reduced electrical conductivity (ECe), and enhanced the effectiveness of gypsum by facilitating the downward movement of displaced sodium ions and soluble salts (Ayers and Westcot, 1985; Rietz and Haynes, 2003). Under straw-amended treatments, irrigation further promoted microbial activity by maintaining favorable soil moisture for straw decomposition and the production of organic binding agents, which strengthened soil aggregation (Liang et al., 2005). Conversely, in plots with insufficient or irregular irrigation, the limited leaching potential may have restricted sodium removal, thereby constraining aggregate stability. The synergistic interaction between irrigation, gypsum, and straw incorporation thus improved both the chemical and physical environments of saline-sodic soils, promoting better organic matter accumulation, greater aggregate stability, and enhanced soil resilience under the semi-arid conditions of the Khipro region.

After a two-year period, water-stable aggregates (WSA) exhibited a statistically significant increase (P ≤ 0.05) in treatments amended with straw compared to those without amendments. This finding aligns with observations by Kantrikrom et al. (2020), who noted that soil organic matter (SOM) enhances the development of larger, more water-stable aggregates, as indicated by an increased mean weight diameter (MWD). The presence of soil organic carbon (SOC) facilitates the binding of smaller aggregates into larger, more stable structures, thereby improving aggregate stability and MWD, consistent with reports from other studies (Emami et al., 2014; Igwe and Stahr, 2004; Mamedov et al., 2007; Ogunwole, 2008).

After a two-year period, treatments incorporating crop straw exhibited a significant increase in aggregate stability (AS) (P ≤ 0.05) compared to non-amended controls. This observation aligns with findings by Barzegar et al. (1997), who noted that incorporating crop residues enhanced soil aggregate stability by elevating soil organic matter (SOM) levels and mitigating soil sodicity. Similarly, Akhtar et al. (2018) demonstrated that straw incorporation significantly boosted the proportion of water-stable macroaggregates, thereby improving overall soil structural integrity. Furthermore, Bruce et al. (1992) identified a positive linear correlation between enhanced aggregate stability and increased soil carbon content. Caron et al. (1992) also reported substantial improvements in soil aggregate stability following organic matter additions. Additional studies, including those by Reid and Goss (1981); Cheny and Swift (1984), and Yazdanpanah et al. (2016), have consistently shown that organic matter applications markedly enhance soil structural stability, particularly in terms of water-stable aggregation. In the current study, shallow tillage treatments (e.g., STWS3, STWS7, STWS10, STCK, and STCTRL) resulted in significantly higher (P ≤ 0.05) soil organic carbon (SOC), water-stable aggregates (WSA), mean weight diameter (MWD), and AS compared to corresponding deep tillage treatments. These findings are consistent with Sommer and De Pauw (2011), who observed that shallow tillage increased SOM in the 0–0.20 m soil layer by an average of 2.7 g kg^−^¹ compared to the 0.20–0.30 m layer across various residue management practices. Vrdoljak and Sposito (2002) further noted that topsoil layers, enriched with organic matter and plant roots, exhibited greater aggregate size distribution, contributing to enhanced soil structural stability, as evidenced by a significant increase in the stability index (SI) (P ≤ 0.01) over five years. Akhter et al. (2004) also reported the most pronounced increase in AS in the topsoil relative to control treatments. The observed increases in SOM, aggregate size, and stability in shallow tillage systems likely stem from reduced organic matter mineralization due to minimal soil disturbance compared to moldboard plowing, as supported by Cannell and Hawes (1994); Aase and Pikul (1995), and Blevins and Frye (1993). Additionally, Kaewmano et al. (2009) found that water-stable aggregate content in salt-affected soils decreased with soil depth, paralleling reductions in clay and organic matter content. This suggests that high sodium levels in such soils compromise aggregate stability, rendering soil structures more susceptible to degradation (Moghimi et al., 2012).

In the current research, significant increases (P ≤ 0.05) in soil organic matter (SOM), water-stable aggregates (WSA), mean weight diameter (MWD), and aggregate stability (AS) were observed with increasing rates of straw incorporation into the soil, following the trend ST_WS10_ > DT_WS10_ > ST_WS7_ > DT_WS7_ > ST_WS3_ > DT_WS3_. These findings align with those of Tan et al. (2007) and Rasmussen and Collins (1991), who demonstrated a strong correlation between SOM content and the quantity of residue applied to the soil. Similarly, Tejada et al. (2006) reported that the addition of organic matter enhanced soil structural stability, with pronounced effects at higher application rates (10 t ha^−^¹) and after an extended experimental duration (5 years). These results are consistent with observations by Chenu et al. (2000); Puget et al. (2000), and Tejada and Gonzalez (2003, 2004), who emphasized that soil structure quality is closely tied to both the quantity and characteristics of organic matter incorporated. This relationship may be attributed to the presence of humic substances and microbial byproducts within soil organic carbon (SOC), as noted by Shepherd (2001) and Piccolo and Mbagwu (1990), which play a critical role in forming clay-organic complexes that enhance soil stability.

Soil enzymes play a critical role in nutrient cycling within agroecosystems, serving as key indicators of soil health and vitality (Zhao et al., 2016). Research has demonstrated that shallow tillage enhances the activity of soil enzymes, such as β-glucosidase, urease, phosphatase, and catalase, particularly within the 0–10 cm soil depth (Kabiri et al., 2016). Findings from a recent study conducted between 2018 and 2019 revealed significant increases in soil enzyme activities, including soil catalase (S-CAT), soil urease (S-URE), soil acid phosphatase (S-APH), and soil cellulase (S-CL), at a depth of 0–15 cm under shallow tillage with straw incorporation (STW10) (**Figure 7**). In contrast, deep tillage practices (DTW10) resulted in modest improvements in S-URE, S-APH, and S-CL activities at depths of 15–30 cm. Notably, S-URE activity was relatively higher at 30–45 cm under DTW10 compared to other enzymes at the same depth (**Figure 8**). These results suggest that shallow tillage (0–15 cm) under STW10 has a more pronounced effect on soil enzyme activities than deeper tillage, whereas deep tillage (DTW10) is more effective at 30–45 cm than at shallower depths. Additionally, the incorporation of straw under STW10 significantly enhanced enzyme activities in the surface soil layer, highlighting the importance of straw management in promoting enzyme secretions essential for straw decomposition and nutrient cycling in soils (López-Garrido et al., 2014).

## Conclusions

The study demonstrated that soil organic matter (SOM) and soil structure parameters such as water-stable aggregates (WSA), mean weight diameter (MWD), and aggregate stability (AS) were significantly greater under straw-incorporated treatments than under treatments without straw after two years. The higher the rate of straw incorporation, the greater the improvements in SOM and soil structural stability. Shallow tillage treatments consistently outperformed deep tillage in enhancing SOM and aggregation, while gypsum application alone showed no significant effect on soil structure. These findings indicate that the incorporation of crop straw into saline-sodic soils gradually enhances SOM and soil structural integrity, with more pronounced effects after two years of continuous application.

## 5 Practical outcomes

From a practical standpoint, the findings provide clear guidance for sustainable soil management in arid and semi-arid regions such as Sindh, Pakistan. Farmers are encouraged to adopt shallow tillage combined with straw incorporation at 7–10 Mg ha^−^¹ to enhance soil aggregation, water infiltration, and nutrient retention while gradually improving soil organic matter and reducing sodicity. This integrated approach reduces dependence on high-cost chemical amendments and supports the long-term restoration of soil fertility and productivity. Furthermore, the outcomes of this research can serve as a scientific basis for land reclamation and agricultural extension programs aimed at improving the resilience and sustainability of salt-affected farmlands.

## Data Availability

The datasets presented in this study can be found in online repositories. The names of the repository/repositories and accession number(s) can be found in the article/**Supplementary Material**.
