# Supplementary material for: Soil organic matter and soil structure changes with tillage practices and straw incorporation in a saline-sodic soil
Source: Front Plant Sci. 2025 Nov 10;16:1681651. doi: 10.3389/fpls.2025.1681651 (PMC12640858; doi:10.3389/fpls.2025.1681651)
Supplement: Supplementary file 1 [file DataSheet1.docx]

**Soil organic matter and soil structure changes with tillage practices and straw incorporation in a saline-sodic soil**

Azhar Ali Laghari^1^, Quart-ul-ain Abro^2^*, Asma Leghari^3^, Akash Kumar^4*^, Lata Kumari^5^, Barkat Ali Nindwani^6^, Sadia Gull^7^

^1^College of Resources Environment, Shanxi Agricultural University, Taigu 030801, Shanxi, China

^2^Business School, University of International Business and Economics, Beijing, 100029, China

^3^Institute of Clean Coal Technology, East China University of Science and Technology, Shanghai, 200237, China

^4^School of Civil Engineering, Guangzhou University, Guangzhou,510006

^5^School of Chemical Engineering of Technology, Tianjin University, Tianjin, 300350, China

^6^Department of Farm Power and Machinery, Faculty of Agricultural Engineering, Sindh Agriculture University, Tandojam 70060, Pakistan

^7^Department of Life Sciences, Western Caspian University, Baku, Azerbaijan;

* Corresponding authors.

Quart-ul-ain Abro

E-mail; [abroqurat@gmail.com](mailto:abroqurat@gmail.com)

Akash Kumar

E-mail; [akash.kumar@gzhu.edu.cn](mailto:akash.kumar@gzhu.edu.cn)

**Appendix A** Analysis of variance (ANOVA) of organic matter and mean weight diameter as influenced by different treatments.

| Parameter | | Mean square | | *F-*value | Prob. |
| --- | --- | --- | --- | --- | --- |
|  |  | Treatment  (d.f. = 25) | Error  (d.f. = 128) |  |  |
| Soil organic matter | Depth  (0-0.15 m) | 0.09483 | 0.00379 | 5.81 | > < 0.0001 |
|  | Depth  (0.16-0.30 m) | 0.07563 | 0.00303 | 5.42 | > < 0.0001 |
|  | Depth  (0.31-0.45 m) | 0.07583 | 0.00303 | 11.11 | > < 0.0001 |
| Water stable aggregates | Unstable aggregates  (<0.25 mm) | 2660.45 | 106.418 | 59.70 | <> 0.0001 |
|  | Stable aggregates  (>0.25 mm) | 2696.99 | 107.880 | 55.15 | <> 0.0001 |
| Mean weight diameter (mm) | | 4.35891 | 0.17436 | 120.38 | <> 0.0001 |
| Aggregate stability (%) | | 633.995 | 25.3598 | 12.55 | <> 0.0001 |

**Appendix B** Analysis of variance (ANOVA) of organic matter as influenced by different treatments. DF =degrees of freedom, MS=mean sum of squares due to the source, F = F statistic, *p* = level of significance, *p* value.

| Source of  variation | Depth | | | | | | | | | | | | | |
| --- | --- | --- | --- | --- | --- | --- | --- | --- | --- | --- | --- | --- | --- | --- |
|  | 0-0.15 m | | | |  | 0.16-0.30 m | | | |  | 0.31-0.45 m | | | |
|  | DF | MS | F | *p* |  | DF | MS | F | *p* |  | DF | MS | F | *p* |
| Treatments | 12 | 0.00501 | 13.23 | <> 0.0001 |  | 12 | 0.00429 | 10.41 | <> 0.0001 |  | 12 | 0.00369 | 14.90 | <> 0.0001 |
| Tillage | 1 | 0.01674 | 44.18 | <> 0.0001 |  | 1 | 0.00255 | 6.19 | 0.0145 |  | 1 | 0.01764 | 71.35 | <> 0.0001 |
| Year | 1 | 0.00659 | 17.39 | < 0.0001 |  | 1 | 0.00135 | 3.28 | 0.0733 |  | 1 | 0.00212 | 8.57 | 0.0042 |
| Tr × Tillage | 12 | 0.00150 | 3.95 | < 0.0001 |  | 12 | 0.00180 | 4.35 | <> 0.0001 |  | 12 | 0.00116 | 4.71 | <> 0.0001 |
| Tr × Year | 12 | 0.00128 | 3.38 | 0.0004 |  | 12 | 0.00137 | 3.33 | 0.0004 |  | 12 | 0.00033 | 1.32 | 0.2214 |
| Tillage × Year | 1 | 0.00038 | 1.01 | 0.3180 |  | 1 | 0.00111 | 2.70 | 0.1032 |  | 1 | 0.00078 | 3.16 | 0.0785 |
| Tillage × Tr × Year | 12 | 0.00188 | 4.96 | >< 0.0001 |  | 12 | 0.00087 | 2.11 | 0.0222 |  | 12 | 0.00024 | 0.99 | 0.4669 |
| Error | 102 | 0.00038 |  |  |  | 102 | 0.00041 |  |  |  | 102 | 0.00025 |  |  |
| Total | 155 |  |  |  |  | 155 |  |  |  |  | 155 |  |  |  |

**Appendix C** Analysis of variance (ANOVA) of water stable aggregates, mean weight diameter and aggregate stability as influenced by different treatments. DF =degrees of freedom, MS=mean sum of squares due to the source, F = F statistic, *p* = level of significance, *p* value.

| Source of variations | Water stable aggregates (%) | | | | | | | | |  | Mean weight diameter (mm) | | | |  | Aggregate Stability (%) | | | |
| --- | --- | --- | --- | --- | --- | --- | --- | --- | --- | --- | --- | --- | --- | --- | --- | --- | --- | --- | --- |
|  | Unstable aggregates (<0.25 mm) | | | |  | Stable aggregates (>0.25 mm) | | | |  |  |  |  |  |  |  |  |  |  |
|  | DF | MS | F | *p* |  | DF | MS | F | *p* |  | DF | MS | F | *p* |  | DF | MS | F | *p* |
| Treatment | 12 | 180.34 | 1345.2 | <> 0.0001 |  | 12 | 183.77 | 619.5 | <> 0.0001 |  | 12 | 0.348 | 2276.5 | <> 0.0001 |  | 12 | 18.59 | 27706.1 | >< 0.0001 |
| Tillage | 1 | 261.56 | 1951.2 | <> 0.0001 |  | 1 | 291.10 | 981.4 | <> 0.0001 |  | 1 | 0.126 | 1116.8 | <> 0.0001 |  | 1 | 385.97 | 575311 | <> 0.0001 |
| Year | 1 | 0.093 | 0.7 | 0.4079 |  | 1 | 0.29 | 1.0 | 0.3270 |  | 1 | 0.001 | 825.9 | <> 0.0001 |  | 1 | 128.57 | 191634 | <> 0.0001 |
| Tr × Tillage | 12 | 19.57 | 146.0 | <> 0.0001 |  | 12 | 16.72 | 56.4 | <> 0.0001 |  | 12 | 0.003 | 7.2 | <> 0.0001 |  | 12 | 2.08 | 3101.8 | <> 0.0001 |
| Tr × Year | 12 | 7.08 | 52.8 | <> 0.0001 |  | 12 | 5.74 | 19.4 | <> 0.0001 |  | 12 | 0.005 | 19.8 | <> 0.0001 |  | 12 | 2.84 | 4234.2 | <> 0.0001 |
| Tillage × Year | 1 | 24.01 | 179.1 | <> 0.0001 |  | 1 | 33.32 | 112.3 | <> 0.0001 |  | 1 | 0.0002 | 29.6 | <> 0.0001 |  | 1 | 67.77 | 101013 | <> 0.0001 |
| Tillage × Tr × Year | 12 | 8.78 | 65.5 | <> 0.0001 |  | 12 | 9.80 | 33.0 | <> 0.0001 |  | 12 | 0.00015 | 1.5 | 0.1519 |  | 12 | 2.35 | 3506.1 | <> 0.0001 |
| Error | 102 | 0.13 |  |  |  | 102 | 0.30 |  |  |  | 102 |  |  |  |  | 102 | 0.001 |  |  |
|  | 155 |  |  |  |  | 155 |  |  |  |  | 155 |  |  |  |  | 155 |  |  |  |

**Appendix D** Soil organic matter under different tillage practices and straw incorporation rates in saline sodic soil.

| **Year** | **Tillage treatments** | | | | | | | | |
| --- | --- | --- | --- | --- | --- | --- | --- | --- | --- |
|  | **Shallow** | | | |  | **Deep** | | | |
|  | **Treatment** | **Sampling depth (m)** | | |  | **Treatment** | **Sampling depth (m)** | | |
|  |  | **0-0.15** | **0.15-0.30** | **0.30-0.45** |  |  | **0-0.15** | **0.15-0.30** | **0.30-0.45** |
| 2018  Soil organic matter content (g hg^-1^) | **ST_CTRL_** | 0.322±0.001^CD^ | 0.322±0.002^FGH^ | 0.325±0.002^D^ |  | **DT_CTRL_** | 0.321±0.001^CD^ | 0.321±0.001^H^ | 0.323±0.001^D^ |
|  | **ST_CK_GR_25_** | 0.324±0.001^CD^ | 0.323±0.001^GH^ | 0.325±0.002^CD^ |  | **DT_CK_GR_25_** | 0.321±0.001^D^ | 0.323±0.001^FGH^ | 0.324±0.001^CD^ |
|  | **ST_CK_GR_50_** | 0.324±0.002^CD^ | 0.322±0.002^FGH^ | 0.330±0.001^CD^ |  | **DT_CK_GR_50_** | 0.321±0.002^CD^ | 0.321±0.001^EFGH^ | 0.326±0.001^CD^ |
|  | **ST_CK_GR_75_** | 0.328±0.001^CD^ | 0.324±0.002^FGH^ | 0.331±0.001^CD^ |  | **DT_CK_GR_75_** | 0.327±0.001^C^ | 0.325±0.001^DEFGH^ | 0.328±0.001^CD^ |
|  | **ST_WS3_GR_25_** | 0.330±0.001^CD^ | 0.329±0.001^EFGH^ | 0.342±0.002^CD^ |  | **DT_WS3_GR_25_** | 0.330±0.001^BC^ | 0.330±0.001^H^ | 0.333±0.001^D^ |
|  | **ST_WS3_GR_50_** | 0.339±0.002^CD^ | 0.336±0.001^DEFGH^ | 0.354±0.002^CD^ |  | **DT_WS3_GR_50_** | 0.335±0.001^CD^ | 0.338±0.001^FGH^ | 0.341±0.001^CD^ |
|  | **ST_WS3_GR_75_** | 0.353±0.001^BC^ | 0.349±0.002^CDEFGH^ | 0.349±0.002^CD^ |  | **DT_WS3_GR_75_** | 0.343±0.001^CD^ | 0.460±0.001^A^ | 0.331±0.001^CD^ |
|  | **ST_WS7_GR_25_** | 0.347±0.001^C^ | 0.345±0.001^CDEFGH^ | 0.363±0.001^ABCD^ |  | **DT_WS7_GR_25_** | 0.333±0.001^CD^ | 0.335±0.001^CDEFGH^ | 0.339±0.001^CD^ |
|  | **ST_WS7_GR_50_** | 0.361±0.002^BC^ | 0.348±0.001^CDEF^ | 0.406±0.002^ABC^ |  | **DT_WS7_GR_50_** | 0.340±0.001^CD^ | 0.339±0.001^CDEF^ | 0.350±0.001^D^ |
|  | **ST_WS7_GR_75_** | 0.404±0.002^AB^ | 0.380±0.001^BCDE^ | 0.359±0.002^CD^ |  | **DT_WS7_GR_75_** | 0.352±0.002^CD^ | 0.356±0.001^FGH^ | 0.338±0.000^D^ |
|  | **ST_WS10_GR_25_** | 0.356±0.001^BC^ | 0.353±0.002^CDEFG^ | 0.372±0.001^ABCD^ |  | **DT_WS10_GR_25_** | 0.339±0.001^CD^ | 0.341±0.001^DEFGH^ | 0.341±0.001^D^ |
|  | **ST_WS10_GR_50_** | 0.371±0.001^A^ | 0.356±0.001^BC^ | 0.434±0.001^A^ |  | **DT_WS10_GR_50_** | 0.342±0.001^CD^ | 0.343±0.001^CDEFGH^ | 0.356±0.001^CD^ |
|  | **ST_WS10_GR_75_** | 0.430±0.001^A^ | 0.404±0.001^B^ | 0.393±0.002^AB^ |  | **DT_WS10_GR_75_** | 0.358±0.002^CD^ | 0.360±0.002^CDEFGH^ | 0.355±0.001^BCD^ |
|  |  |  |  |  |  |  |  |  |  |
| 2019  Soil organic matter content (g hg^-1^) | **ST_CTRL_** | 0.325±0.001^c^ | 0.325±0.001^b^ | 0.330±0.001^d^ |  | **DT_CTRL_** | 0.325±0.007^c^ | 0.325±0.001^b^ | 0.327±0.001^d^ |
|  | **ST_CK_GR_25_** | 0.329±0.002^c^ | 0.328±0.001^b^ | 0.330±0.001^bcd^ |  | **DT_CK_GR_25_** | 0.325±0.001^bc^ | 0.327±0.002^ab^ | 0.328±0.001^bcd^ |
|  | **ST_CK_GR_50_** | 0.329±0.002^abc^ | 0.327±0.002^ab^ | 0.336±0.002^bcd^ |  | **DT_CK_GR_50_** | 0.325±0.000^abc^ | 0.325±0.001^ab^ | 0.331±0.001^bcd^ |
|  | **ST_CK_GR_75_** | 0.334±0.001^ab^ | 0.329±0.002^ab^ | 0.340±0.001^abc^ |  | **DT_CK_GR_75_** | 0.332±0.001^abc^ | 0.330±0.001^ab^ | 0.338±0.001^bcd^ |
|  | **ST_WS3_GR_25_** | 0.339±0.001^a^ | 0.338±0.001^ab^ | 0.352±0.003^d^ |  | **DT_WS3_GR_25_** | 0.341±0.001^abc^ | 0.341±0.002^ab^ | 0.345±0.001^bcd^ |
|  | **ST_WS3_GR_50_** | 0.350±0.001^c^ | 0.347±0.004^b^ | 0.369±0.001^cd^ |  | **DT_WS3_GR_50_** | 0.347±0.001^c^ | 0.350±0.001^b^ | 0.355±0.001^cd^ |
|  | **ST_WS3_GR_75_** | 0.368±0.001^abc^ | 0.364±0.001^ab^ | 0.360±0.001^bcd^ |  | **DT_WS3_GR_75_** | 0.357±0.001^abc^ | 0.360±0.001^ab^ | 0.342±0.002^bcd^ |
|  | **ST_WS7_GR_25_** | 0.358±0.001^abc^ | 0.356±0.001^ab^ | 0.377±0.001^bc^ |  | **DT_WS7_GR_25_** | 0.344±0.002^abc^ | 0.346±0.001^ab^ | 0.351±0.001^bcd^ |
|  | **ST_WS7_GR_50_** | 0.375±0.001^abc^ | 0.361±0.002^ab^ | 0.427±0.001^bcd^ |  | **DT_WS7_GR_50_** | 0.352±0.004^abc^ | 0.351±0.002^ab^ | 0.370±0.001^cd^ |
|  | **ST_WS7_GR_75_** | 0.425±0.002^c^ | 0.400±0.001^ab^ | 0.374±0.001^cd^ |  | **DT_WS7_GR_75_** | 0.372±0.001^c^ | 0.376±0.001^b^ | 0.351±0.001^cd^ |
|  | **ST_WS10_GR_25_** | 0.372±0.002^abc^ | 0.369±0.001^ab^ | 0.390±0.001^bcd^ |  | **DT_WS10_GR_25_** | 0.352±0.000^abc^ | 0.354±0.001^ab^ | 0.355±0.001^bcd^ |
|  | **ST_WS10_GR_50_** | 0.389±0.001^bc^ | 0.373±0.001^ab^ | 0.459±0.001^ab^ |  | **DT_WS10_GR_50_** | 0.356±0.002^abc^ | 0.357±0.001^ab^ | 0.378±0.001^bcd^ |
|  | **ST_WS10_GR_75_** | 0.454±0.002^a^ | 0.429±0.001^a^ | 0.421±0.001^a^ |  | **DT_WS10_GR_75_** | 0.380±0.001^abc^ | 0.382±0.001^ab^ | 0.376±0.001^bcd^ |

Note:

^1^ST_CTRL_ control treatment under shallow tillage; ST_CK_GR_25_ conventional tillage under shallow tillage at 25% gypsum requirement (GR); ST_CK_GR_50_ conventional tillage under shallow tillage at 50% gypsum requirement (GR); ST_CK_GR_75_ conventional tillage under shallow tillage at 75% gypsum requirement (GR); ST_WS3_GR_25_ straw incorporation at the rate of 3 Mg ha^-1^ under shallow tillage at 25% gypsum requirement (GR); ST_WS3_GR_50_ straw incorporation at the rate of 3 Mg ha^-1^ under shallow tillage at 50% gypsum requirement (GR); ST_WS3_GR_75_ straw incorporation at the rate of 3 Mg ha^-1^ under shallow tillage at 75% gypsum requirement (GR); ST_WS7_GR_25_ straw incorporation at the rate of 7 Mg ha^-1^ under shallow tillage at 25% gypsum requirement (GR); ST_WS7_GR_50_ straw incorporation at the rate of 7 Mg ha^-1^ under shallow tillage at 50% gypsum requirement (GR); ST_WS7_GR_75_ straw incorporation at the rate of 7 Mg ha^-1^ under shallow tillage at 75% gypsum requirement (GR); ST_WS10_GR_25_ straw incorporation at the rate of 10 Mg ha^-1^ under shallow tillage at 25% gypsum requirement (GR); ST_WS10_GR_50_ straw incorporation at the rate of 10 Mg ha^-1^ under shallow tillage at 50% gypsum requirement (GR); ST_WS10_GR_75_ straw incorporation at the rate of 10 Mg ha^-1^ under shallow tillage at 75% gypsum requirement (GR).

^2^DT_CTRL_ control treatment under deep tillage; DT_CK_GR_25_ conventional tillage under deep tillage at 25% gypsum requirement (GR); DT_CK_GR_50_ conventional tillage under deep tillage at 50% gypsum requirement (GR); DT_CK_GR_75_ conventional tillage under deep tillage at 75% gypsum requirement (GR); DT_WS3_GR_25_ straw incorporation at the rate of 3 Mg ha^-1^ under deep tillage at 25% gypsum requirement (GR); DT_WS3_GR_50_ straw incorporation at the rate of 3 Mg ha^-1^ under deep tillage at 50% gypsum requirement (GR); DT_WS3_GR_75_ straw incorporation at the rate of 3 Mg ha^-1^ under deep tillage at 75% gypsum requirement (GR); DT_WS7_GR_25_ straw incorporation at the rate of 7 Mg ha^-1^ under deep tillage at 25% gypsum requirement (GR); DT_WS7_GR_50_ straw incorporation at the rate of 7 Mg ha^-1^ under deep tillage at 50% gypsum requirement (GR); DT_WS7_GR_75_ straw incorporation at the rate of 7 Mg ha^-1^ under deep tillage at 75% gypsum requirement (GR); DT_WS10_GR_25_ straw incorporation at the rate of 10 Mg ha^-1^ under deep tillage at 25% gypsum requirement (GR); DT_WS10_GR_50_ straw incorporation at the rate of 10 Mg ha^-1^ under deep tillage at 50% gypsum requirement (GR); DT_WS10_GR_75_ straw incorporation at the rate of 10 Mg ha^-1^ under deep tillage at 75% gypsum requirement (GR).

^3^Values followed by the same uppercase letter are not significantly different across depth and/or treatment according to Duncan’s multiple range test (*P*≤0.05) within the same year (2018).

^4^Values followed by the same lowercase letter are not significantly different across depth and/or treatment according to Duncan’s multiple range test (*P*≤0.05) within the same year (2019).

^5^Mean ± standard error.

**Appendix E** Water stable aggregates under different tillage practices and straw incorporation rates in saline sodic soil.

| **Year** | **Type of tillage** | | | | | | |
| --- | --- | --- | --- | --- | --- | --- | --- |
|  | **Shallow** | | |  | **Deep** | | |
|  | **Treatment** | **Aggregate Type** | |  | **Treatment** | **Aggregate Type** | |
|  |  | **Unstable**  **< 0.25 mm** | **Stable**  **> 0.25 mm** | **......** |  | **Unstable**  **< 0.25 mm** | **Stable**  **> 0.25 mm** |
| 2018  Water stable aggregates | **ST_CTRL_** | 22.2±0.22^GH^ | 77.8±0.3^HIJ^ |  | **DT_CTRL_** | 30.2±0.129^A^ | 69.8±0.168^O^ |
|  | **ST_CK_GR_25_** | 24.57±0.129^C^ | 75.4±0.091^M^ |  | **DT_CK_GR_25_** | 26.4±0.238^B^ | 73.6±0.173^NJ^ |
|  | **ST_CK_GR_50_** | 23.96±0.5^CD^ | 76±0.235^JKLM^ |  | **DT_CK_GR_50_** | 23.2±0.147^EFG^ | 76.7±0.147^JKLM^ |
|  | **ST_CK_GR_75_** | 24.14±0.108^CDE^ | 75.9±0.108^KLM^ |  | **DT_CK_GR_75_** | 24.1±0.082^CDE^ | 75.9±0.158^KLM^ |
|  | **ST_WS3_GR_25_** | 21.965±0.314^GH^ | 78±0.258^FGH^ |  | **DT_WS3_GR_25_** | 24.2±0.158^DEF^ | 75.8±0.227^LM^ |
|  | **ST_WS3_GR_50_** | 22.635±0.274^G^ | 77.4±0.129^IJ^ |  | **DT_WS3_GR_50_** | 22.8±0.158^FG^ | 77.3±0.071^IJKL^ |
|  | **ST_WS3_GR_75_** | 20.18±0.108^I^ | 79.8±0.212^EFG^ |  | **DT_WS3_GR_75_** | 23.5±0.168^DEF^ | 76.6±0.129^JKLM^ |
|  | **ST_WS7_GR_25_** | 16.6±0.238^K^ | 83.4±0.091^CD^ |  | **DT_WS7_GR_25_** | 19.2±0.091^J^ | 80.8±0.122^EFG^ |
|  | **ST_WS7_GR_50_** | 15.76±0.091^L^ | 84.2±0.091^BC^ |  | **DT_WS7_GR_50_** | 21.4±0.108^H^ | 78.6±0.108^GHI^ |
|  | **ST_WS7_GR_75_** | 13.9±0.227^MN^ | 86.1±0.204^A^ |  | **DT_WS7_GR_75_** | 22.5±0.183^G^ | 77.5±0.196^IJK^ |
|  | **ST_WS10_GR_25_** | 14.48±0.414^M^ | 85.5±0.108^AB^ |  | **DT_WS10_GR_25_** | 18.9±0.196^J^ | 81.1±0.091^E^ |
|  | **ST_WS10_GR_50_** | 14.345±0.122^MN^ | 85.6±0.147^AB^ |  | **DT_WS10_GR_50_** | 19.3±0.091^IJ^ | 80.7±0.158^EF^ |
|  | **ST_WS10_GR_75_** | 13.64±0.158^N^ | 86.4±0.178^A^ |  | **DT_WS10_GR_75_** | 16.9±0.196^J^ | 83.1±0.108^D^ |
| 2018  Water stable aggregates | **ST_CTRL_** | 27.8±0.091^ab^ | 72.2±0.196^op^ |  | **DT_CTRL_** | 29.2±0.173^a^ | 70.8±0.342^p^ |
|  | **ST_CK_GR_25_** | 25.7±0.204^de^ | 74.3±0.082^mn^ |  | **DT_CK_GR_25_** | 25.4±0.147^de^ | 74.6±0.147^mn^ |
|  | **ST_CK_GR_50_** | 26.1±0.147^cd^ | 74±0.158^mno^ |  | **DT_CK_GR_50_** | 25.2±0.108^e^ | 74.9±0.227^lm^ |
|  | **ST_CK_GR_75_** | 23.5±0.108^f^ | 76.5±0.091^kl^ |  | **DT_CK_GR_75_** | 27.5±0.187^bc^ | 72.5±0.147^no^ |
|  | **ST_WS3_GR_25_** | 22.7±0.204^f^ | 77.3±0.147^jk^ |  | **DT_WS3_GR_25_** | 20.2±0.091^gh^ | 79.8±0.158^ghi^ |
|  | **ST_WS3_GR_50_** | 23.2±0.212^f^ | 76.9±0.22^k^ |  | **DT_WS3_GR_50_** | 20.5±0.091^g^ | 79.6±0.147^hi^ |
|  | **ST_WS3_GR_75_** | 18.4±0.129^ij^ | 81.6±0.147^efgh^ |  | **DT_WS3_GR_75_** | 19.2±0.082^hi^ | 90.8±0.173^ghi^ |
|  | **ST_WS7_GR_25_** | 16±0.311l^m^ | 84±0.158^b^ |  | **DT_WS7_GR_25_** | 20.7±0.159^g^ | 79.3±0.091^ij^ |
|  | **ST_WS7_GR_50_** | 17.5±0.158^jk^ | 82.5±0.227^de^ |  | **DT_WS7_GR_50_** | 16.9±0.108^kl^ | 83.1±0.091^cde^ |
|  | **ST_WS7_GR_75_** | 16.2±0.158^l^ | 83.8±0.168^bcd^ |  | **DT_WS7_GR_75_** | 19.9±0.122^gh^ | 80.1±0.342^fghi^ |
|  | **ST_WS10_GR_25_** | 15.5±0.147^lm^ | 84.5±0.248^bc^ |  | **DT_WS10_GR_25_** | 17.6±0.147^gh^ | 82.4±0.129^def^ |
|  | **ST_WS10_GR_50_** | 14.7±0.14^1m^ | 85.3±0.158^b^ |  | **DT_WS10_GR_50_** | 20.2±0.122^gh^ | 79.8±0.196^ghi^ |
|  | **ST_WS10_GR_75_** | 12.6±0.122^n^ | 87.4±0.091^a^ |  | **DT_WS10_GR_75_** | 18.3±0.122^ij^ | 81.7±0.071^efg^ |

Note:

^1^Refer to Table 4 for the treatment abbreviations.^2^Values followed by the same uppercase letter are not significantly different across depth and/or treatment according to Duncan’s multiple range test (*P*≤0.05) within the same year (2018).

^3^Values followed by the same lowercase letter are not significantly different across depth and/or treatment according to Duncan’s multiple range test (*P*≤0.05) within the same year (2019).

^4^Mean ± standard error.

**Appendix F** Mean weight diameter (%) under different tillage practices and straw incorporation rates in saline sodic soil.

|  | **Shallow Tillage** | |  | **Deep Tillage** | |
| --- | --- | --- | --- | --- | --- |
| **Treatment** | **2018** | **2019** | **Treatment** | **2018** | **2019** |
| **ST_CTRL_** | 1.445±0.012^Q^ | 1.485±0.019 ^p^ | **DT_CTRL_** | 1.4±0.014  ^O^ | 1.43±0.0145 ^o^ |
| **ST_CK_GR_25_** | 1.53±0.0125^O^ | 1.58±0.013 ^mn^ | **DT_CK_GR_25_** | 1.505±0.012 ^N^ | 1.535±0.014 ^mn^ |
| **ST_CK_GR_50_** | 1.54±0.0115^O^ | 1.6±0.0155 ^m^ | **DT_CK_GR_50_** | 1.525±0.0125 ^MN^ | 1.555±0.0145 ^m^ |
| **ST_CK_GR_75_** | 1.55±0.012^NO^ | 1.61±0.012 ^lm^ | **DT_CK_GR_75_** | 1.535±0.014 ^MN^ | 1.57±0.014 ^m^ |
| **ST_WS3_GR_25_** | 1.64±0.0175 ^KL^ | 1.735±0.011 ^j^ | **DT_WS3_GR_25_** | 1.63±0.02 ^L^ | 1.69±0.008 ^jk^ |
| **ST_WS3_GR_50_** | 1.65±0.012 ^K^ | 1.755±0.0135 ^j^ | **DT_WS3_GR_50_** | 1.635±0.0135 ^L^ | 1.7±0.0105 ^ijk^ |
| **ST_WS3_GR_75_** | 1.675±0.013 ^K^ | 1.775±0.017 ^j^ | **DT_WS3_GR_75_** | 1.655±0.018 ^KL^ | 1.72±0.0105 ^hij^ |
| **ST_WS7_GR_25_** | 1.81±0.012 ^I^ | 1.885±0.012 ^fg^ | **DT_WS7_GR_25_** | 1.74±0.018 ^GHI^ | 1.795±0.011 ^ef^ |
| **ST_WS7_GR_50_** | 1.835±0.011 ^HI^ | 1.9±0.0135 ^ef^ | **DT_WS7_GR_50_** | 1.76±0.013 ^FGH^ | 1.82±0.01 ^de^ |
| **ST_WS7_GR_75_** | 1.865±0.01 ^GH^ | 1.93±0.0165 ^cde^ | **DT_WS7_GR_75_** | 1.77±0.0135 ^FG^ | 1.85±0.009 ^cd^ |
| **ST_WS10_GR_25_** | 1.92±0.01 ^DE^ | 2.015±0.0125 ^b^ | **DT_WS10_GR_25_** | 1.815±0.0125 ^DE^ | 1.895±0.011 ^b^ |
| **ST_WS10_GR_50_** | 1.94±0.0145 ^CD^ | 2.03±0.0325 ^ab^ | **DT_WS10_GR_50_** | 1.85±0.012 ^CD^ | 1.925±0.0155 ^ab^ |
| **ST_WS10_GR_75_** | 1.955±0.0085 ^C^ | 2.06±0.0215 ^a^ | **DT_WS10_GR_75_** | 1.89±0.0155 ^BC^ | 1.955±0.0125 ^a^ |

^1^Refer to Table 4 for the treatment abbreviations.

^2^Values followed by the same uppercase letter are not significantly different across depth and/or treatment according to Duncan’s multiple range test (*P*≤0.05) within the same year (2018).

^3^Values followed by the same lowercase letter are not significantly different across depth and/or treatment according to Duncan’s multiple range test (*P*≤0.05) within the same year (2019).

^4^Mean ± standard error.

**Appendix G** Aggregate stability under different tillage practices and straw incorporation rates in saline sodic soil.

|  | **Shallow Tillage** | |  | **Deep Tillage** | |
| --- | --- | --- | --- | --- | --- |
| **Treatment** | **2018** | **2019** | **Treatment** | **2018** | **2019** |
| **ST_CTRL_** | 2.0842±0.135 ^S^ | 2.5054±0.15 ^l^ | **DT_CTRL_** | 1.9474±0.14 ^S^ | 2.368±0.16 ^p^ |
| **ST_CK_GR_25_** | 2.2758±0.1311 ^R^ | 2.6247±0.25 ^k^ | **DT_CK_GR_25_** | 2.0323±0.181 ^S^ | 2.6053±0.181 ^n^ |
| **ST_CK_GR_50_** | 2.487±0.108 ^R^ | 2.9256±0.096 ^j^ | **DT_CK_GR_50_** | 2.3635±0.15 ^R^ | 2.6034±0.171 ^k^ |
| **ST_CK_GR_75_** | 2.615±0.129 ^Q^ | 2.97±0.098 ^f^ | **DT_CK_GR_75_** | 2.5444±0.139 ^R^ | 2.7815±0.19 ^j^ |
| **ST_WS3_GR_25_** | 2.6861±0.0913 ^Q^ | 3.38±0.122 ^g^ | **DT_WS3_GR_25_** | 2.5924±0.151 ^O^ | 3.0484±0.16 ^i^ |
| **ST_WS3_GR_50_** | 2.811±0.191 ^P^ | 3.7036±0.21 ^e^ | **DT_WS3_GR_50_** | 2.6987±0.18 ^M^ | 3.2112±0.14 ^g^ |
| **ST_WS3_GR_75_** | 2.935±0.168 ^NO^ | 3.84±0.212 ^b^ | **DT_WS3_GR_75_** | 2.8048±0.179 ^L^ | 3.4128±0.17 ^d^ |
| **ST_WS7_GR_25_** | 3.226±0.214 ^P^ | 3.894±0.218 ^h^ | **DT_WS7_GR_25_** | 3.0974±0.14 ^Q^ | 3.1918±0.2 ^h^ |
| **ST_WS7_GR_50_** | 3.394±0.167 ^OP^ | 4.7908±0.188 ^d^ | **DT_WS7_GR_50_** | 3.1126±0.16 ^O^ | 3.8974±0.23 ^i^ |
| **ST_WS7_GR_75_** | 3.465±0.1472 ^N^ | 5.3051±0.1707 ^c^ | **DT_WS7_GR_75_** | 3.3044±0.11 ^M^ | 4.3061±0.18 ^e^ |
| **ST_WS10_GR_25_** | 4.278±0.19129 ^OP^ | 5.745±0.177 ^i^ | **DT_WS10_GR_25_** | 3.4532±0.15 ^E^ | 4.8183±0.15 ^e^ |
| **ST_WS10_GR_50_** | 4.588±0.0915 ^N^ | 7.4423±0.176 ^b^ | **DT_WS10_GR_50_** | 3.9167±0.13 ^C^ | 5.008±0.17 ^c^ |
| **ST_WS10_GR_75_** | 5.034±0.11 ^M^ | 9.21±0.1912 ^a^ | **DT_WS10_GR_75_** | 4.22±0.14 ^B^ | 6.6226±0.14 ^b^ |

^1^Refer to Table 4 for the treatment abbreviations.

^2^Values followed by the same uppercase letter are not significantly different across depth and/or treatment according to Duncan’s multiple range test (*P*≤0.05) within the same year (2018).

^3^Values followed by the same lowercase letter are not significantly different across depth and/or treatment according to Duncan’s multiple range test (*P*≤0.05) within the same year (2019).

^4^Mean ± standard error.
